# A Meta-Analysis of Brain DNA Methylation Across Sex, Age, and Alzheimer's Disease Points for Accelerated Epigenetic Aging in Neurodegeneration

**DOI:** 10.3389/fnagi.2021.639428

**Published:** 2021-03-11

**Authors:** Camilla Pellegrini, Chiara Pirazzini, Claudia Sala, Luisa Sambati, Igor Yusipov, Alena Kalyakulina, Francesco Ravaioli, Katarzyna M. Kwiatkowska, Danielle F. Durso, Mikhail Ivanchenko, Daniela Monti, Raffaele Lodi, Claudio Franceschi, Pietro Cortelli, Paolo Garagnani, Maria Giulia Bacalini

**Affiliations:** ^1^Istituto di Ricovero e Cura a Carattere Scientifico Istituto delle Scienze Neurologiche di Bologna, Bologna, Italy; ^2^Department of Physics and Astronomy, University of Bologna, Bologna, Italy; ^3^Department of Biomedical and Neuromotor Sciences, University of Bologna, Bologna, Italy; ^4^Institute of Information Technologies, Mathematics and Mechanics, Lobachevsky University, Nizhny Novgorod, Russia; ^5^Department of Experimental, Diagnostic and Specialty Medicine, University of Bologna, Bologna, Italy; ^6^Department of Neurology, University of Massachusetts Medical School, Worcester, MA, United States; ^7^Department of Experimental and Clinical Biomedical Sciences “Mario Serio,” University of Florence, Florence, Italy; ^8^Department of Laboratory Medicine, Clinical Chemistry, Karolinska Institutet, Karolinska University Hospital, Stockholm, Sweden; ^9^Applied Biomedical Research Center, Policlinico S.Orsola-Malpighi Polyclinic, Bologna, Italy; ^10^National Research Council of Italy Institute of Molecular Genetics “Luigi Luca Cavalli-Sforza,” Unit of Bologna, Bologna, Italy

**Keywords:** DNA methylation, Alzheimer's disease, brain, sex, aging

## Abstract

Alzheimer's disease (AD) is characterized by specific alterations of brain DNA methylation (DNAm) patterns. Age and sex, two major risk factors for AD, are also known to largely affect the epigenetic profiles in brain, but their contribution to AD-associated DNAm changes has been poorly investigated. In this study we considered publicly available DNAm datasets of four brain regions (temporal, frontal, entorhinal cortex, and cerebellum) from healthy adult subjects and AD patients, and performed a meta-analysis to identify sex-, age-, and AD-associated epigenetic profiles. In one of these datasets it was also possible to distinguish 5-methylcytosine (5mC) and 5-hydroxymethylcytosine (5hmC) profiles. We showed that DNAm differences between males and females tend to be shared between the four brain regions, while aging differently affects cortical regions compared to cerebellum. We found that the proportion of sex-dependent probes whose methylation is modified also during aging is higher than expected, but that differences between males and females tend to be maintained, with only a few probes showing age-by-sex interaction. We did not find significant overlaps between AD- and sex-associated probes, nor disease-by-sex interaction effects. On the contrary, we found that AD-related epigenetic modifications are significantly enriched in probes whose DNAm varies with age and that there is a high concordance between the direction of changes (hyper or hypo-methylation) in aging and AD, supporting accelerated epigenetic aging in the disease. In summary, our results suggest that age-associated DNAm patterns concur to the epigenetic deregulation observed in AD, providing new insights on how advanced age enables neurodegeneration.

## Introduction

Alzheimer's disease (AD) is a chronic neurodegenerative disease that leads to a progressive decay of cognitive abilities and self-sufficiency. Neuronal loss involves multiple brain regions that are progressively affected by the disease. Hippocampus and entorhinal cortex exhibit the earliest pathological changes, preceding the onset of clinical signs and cognitive impairment by several years, and later the disease spreads to the other brain regions (Braak and Braak, 1991; Van Hoesen et al., 1991; Scahill et al., 2002; Coupé et al., 2019).

Advanced age and female sex are the two major non-modifiable risk factors for AD (Hickman et al., 2016; Podcasy and Epperson, 2016; Fisher et al., 2018). More than 95% of cases of AD occur after 65 years of age (late onset AD), and AD prevalence increases exponentially between 65 and 85 years (Hebert et al., 1995; Kawas and Corrada, 2006). Two-thirds of clinically diagnosed cases of AD are women, and the fact that women live longer than man does not fully explain this sex bias for AD (Pike, 2017; Nebel et al., 2018).

The etiology and pathogenesis of AD are complex and likely result from the interplay between genetic and environmental factors during lifespan. In this scenario epigenetic modifications have attracted increased interest in the study of AD, as they integrate genetic background and environment and modulate genomic organization and gene expression. Epigenetic modifications regulate brain biology throughout development and lifetime, influencing neuronal plasticity, cognition, and behavior (Fagiolini et al., 2009), and deregulation of brain epigenetic patterns has been associated to the pathogenesis of neurological and psychiatric disorders (Landgrave-Gómez et al., 2015; Jaffe et al., 2016). Several studies in post-mortem AD brains have investigated the role of DNA methylation (DNAm), the best-characterized epigenetic modification, identifying a number of CpG sites that show robust changes in DNAm compared to non-demented controls (Lunnon et al., 2014; Gasparoni et al., 2018; Smith et al., 2018, 2019, 2020; Altuna et al., 2019; Lardenoije et al., 2019; Semick et al., 2019; Smith R. G. et al., 2020; Wei et al., 2020).

Interestingly, the two major non-modifiable AD risk factors mentioned above, i.e., sex and age, are also among the main biological variables that influence epigenetic patterns in most human tissues, including brain (Gilbert et al., 2019).

Genome-wide DNAm differences between males and females have been found in whole blood (Singmann et al., 2015) and have been related to the sex-biased risk of psychiatric diseases (Maschietto et al., 2017). A similar link has been reported also in brain (Xia et al., 2019) where sex-specific DNAm patterns are established early during prenatal development (Spiers et al., 2015; Perzel Mandell et al., 2020) and are at least in part maintained in the adulthood (Xu et al., 2014; Spiers et al., 2015), contributing to the profound differences in brain functions between males and females (McCarthy et al., 2009; Forger, 2016; Gegenhuber and Tollkuhn, 2019) and to the different onset of psychiatric disorders (Perzel Mandell et al., 2020).

DNAm patterns are largely remodeled during aging (Pal and Tyler, 2016), where a trend toward global loss of DNAm together with hypermethylation at specific loci is observed (Xiao et al., 2019). Although with some differences among brain regions (Hernandez et al., 2011; Horvath et al., 2015), age-associated epigenetic changes interest also the brain, likely contributing to the structural and functional alterations that can result in progressive cognitive decline and increased susceptibility to neurodegenerative disorders (Bishop et al., 2010; Lardenoije et al., 2015).

So far, only few studies have considered how sex and age interact during lifespan in shaping the epigenome. Data on whole blood indicate that sex-dependent DNAm is remodeled during aging (McCartney et al., 2019), and we suggested that these changes occur at different extent in human models of successful and unsuccessful aging (Yusipov et al., 2020). In mouse hippocampus and human frontal cortex, Masser et al. identified both CpGs in which sex-dependent DNAm is maintained during lifetime, and CpG sites that are differentially affected by aging in relation to sex (Masser et al., 2017). Interestingly, some studies employing epigenetic clocks, i.e., DNAm-based predictors of age, reported accelerated aging in whole blood from males compared to females (Horvath et al., 2016; Xiao et al., 2018; Tajuddin et al., 2019), and the same trend was observed also in brain (Horvath et al., 2016).

Collectively, the available data sustain the importance of sex and aging in shaping the brain epigenome, but so far only one study combined different datasets to identify reproducible sex-associated DNAm profiles (Xia et al., 2019). No study has systematically analyzed multiple datasets and brain regions to identify DNAm patterns resulting from the interaction of sex and age during lifespan, and most importantly no study has evaluated whether sex- and age-dependent DNAm can contribute to epigenetic deregulation in AD, despite the pivotal role of these two factors in AD etiology and pathogenesis.

To fill this gap, in the present paper we performed a meta-analysis of DNAm across sex, age, and AD considering publicly available datasets from different brain regions.

## Materials and Methods

### Datasets

To select DNAm datasets based on Infinium BeadChip technology, the Gene Expression Omnibus (GEO) repository (Clough and Barrett, 2016) was interrogated by the *GEOmetadb* Bioconductor package using the following search terms: “GPL13534,” “GPL21145,” to include only datasets based on the Illumina Infinium HumanMethylation450 and MethylationEPIC BeadChips; “sex,” “gender,” “female,” to include only datasets in which the information on the sex of the subjects was available; “age,” to include only datasets in which the information on the age of the subjects was available; “brain,” “cortex,” “gyrus,” “lobe,” “gray,” to select datasets in which brain samples were analyzed; “control,” “normal,” “non-tumor,” “health,” or “Alzheimer,” “AD,” “Braak,” to select datasets including healthy and AD subjects, respectively. We considered only datasets including more than 10 healthy subjects. As to June 30th 2020, only Illumina Infinium HumanMethylation450 datasets were retrieved.

For the meta-analysis of sex- and age-dependent DNAm in healthy subjects, we selected only datasets including at least 10 males and 10 females, having more than 19 years and spanning an age range of at least 30 years. We further considered only brain regions for which at least two datasets were available. This resulted in eight datasets covering four regions: Frontal cortex (FC), Temporal cortex (TC), Entorhinal cortex (ERC), Cerebellum (CRB) (Table 1).

**Table 1:** Characteristics of the Infinium450k datasets including healthy subjects selected in the present study for the meta-analysis of sex- and age-associated DNAm.

**Number of GEO accession**	**Regions**	**Number of subjects**	**Sex (F/M)**	**Age range (years)**
GSE105109	Entorhinal cortex	27	13/14	58–99
	Cerebellum	28	14/14	58–99
GSE125895	Frontal cortex	47	19/28	51.83–83.64
	Entorhinal cortex	49	20/29	51.83–83.64
GSE134379	Temporal cortex	179	76/103	63–103
	Cerebellum	179	76/103	63–103
GSE59685	Frontal cortex	24	12/12	55–95
	Temporal cortex	26	13/13	40–95
	Cerebellum	23	10/13	40–95
GSE74193	Frontal cortex	216	68/148	19.26–85.2
GSE64509	Frontal cortex	40	22/18	32–114
	Cerebellum	31	21/10	38–114
GSE66351	Frontal cortex	25	10/15	46–88
	Temporal cortex	25	10/15	46–88

For the meta-analysis of AD-associated methylation patterns, we selected only the datasets including subjects over 65 years of age with at least 3 males and 3 females in the control and AD groups. This resulted in eight datasets covering the same brain regions indicated above (Table 2).

**Table 2:** Characteristics of the Infinium450k datasets investigated in the present study including AD patients and non-demented control subjects.

**Number of GEO accession**	**Regions**	**Number of subjects**	**Sex (F/M)**	**Age range (years)**
GSE105109	Entorhinal cortex Ctrl	24	13/11	66–99
	Entorhinal cortex AD	61	27/34	67–97
	Cerebellum Ctrl	25	13/12	66–99
	Cerebellum AD	64	27/37	67–97
GSE125895	Frontal cortex Ctrl	11	5/6	65.04–83.64
	Frontal cortex AD	18	9/9	71.47–92.29
	Entorhinal cortex Ctrl	12	5/7	65.04–83.64
	Entorhinal cortex AD	17	10/7	71.47–92.29
	Cerebellum Ctrl	8	4/4	65.04–83.64
	Cerebellum AD	20	11/8	71.47–92.29
GSE134379	Temporal cortex Ctrl	175	76/99	68–103
	Temporal cortex AD	217	117/100	66–102
	Cerebellum Ctrl	175	74/95	68–103
	Cerebellum AD	217	117/100	66–102
GSE59685	Frontal cortex Ctrl	21	10/11	66–95
	Frontal cortex AD	60	39/21	66–103
	Temporal cortex Ctrl	22	11/11	66–95
	Temporal cortex AD	61	40/21	66–103
	Entorhinal cortex Ctrl	19	8/11	66–95
	Entorhinal cortex AD	58	19/13	66–95
	Cerebellum Ctrl	19	8/11	66–95
	Cerebellum AD	60	39/21	66–103
GSE66351	Frontal cortex Ctrl	12	8/4	71–88
	Frontal cortex AD	35	22/13	67–97
	Temporal cortex Ctrl	12	8/4	71–88
	Temporal cortex AD	37	23/14	67–97
GSE76105	Temporal cortex Ctrl	34	18/16	66–94
	Temporal cortex AD	34	17/17	66–92
GSE80970	Frontal cortex Ctrl	68	34/34	70–108
	Frontal cortex AD	74	54/30	72–103
	Temporal cortex Ctrl	70	36/34	70–108
	Temporal cortex AD	74	54/30	72–103
GSE109627	Temporal cortex Ctrl	36	19/17	73–94
	Temporal cortex AD	46	24/22	70–95

*Ctrl, healthy subjects; AD, Alzheimer's disease patients*.

### Pre-processing

As raw intensities files were not available for some datasets, all the analyses were performed on pre-processed methylation data downloaded from GEO. Potentially ambiguous probes (cross-reactive probes and probes including SNPs; Zhou et al., 2017) were excluded from the analyses. Probes mapping on sex chromosomes were removed, except when the comparison between AD and healthy controls was performed in males and females separately. GSE134379, GSE125895, GSE66351, and GSE76105 did not include probes mapping on sex chromosomes in the pre-processed data downloaded from GEO.

In each dataset, neuron/glia proportions were estimated using Horvath's calculator (Horvath, 2013) which implements the algorithm developed by Guintivano et al. (2013).

For the analysis of 5-methylcytosine (5mC), 5-hydroxymethylcytosine (5hmC), and unmethylated cytosine (5uC) in the GSE105109 dataset, we considered only the samples for which both bisulfite (BS) and oxidative bisulfite (oxBS) were available. ERC included 25 healthy subjects (12 females and 13 males) and 57 AD (25 females and 32 males), while CRB included 28 healthy subjects (14 females and 14 males) and 63 AD (26 females and 37 males). OxBS beta values correspond to 5mC levels; 5hmC levels were calculated by subtracting oxBS beta values from BS beta values (BS-oxBS), while 5uC levels were calculated by subtracting BS beta values from 1 (1-BS; Lardenoije et al., 2019). Negative values returning from the difference BS-oxBS were set to a value close to zero (1 × 10^−7^; Ringh et al., 2019).

### Differential Analysis and Meta-Analysis

To identify differentially methylated positions (DMPs), the *lmFit* function implemented in *limma* R package (Ritchie et al., 2015) was used to fit a linear model to each microarray probe, expressing DNAm as M-values. Association with age was calculated using age as a continuous value and correcting for sex and neuron/glia proportion. Association with sex was calculated using sex as a categorical value and correcting for age and neuron/glia proportion. Association with AD was calculated using AD as a categorical value and correcting for age, sex and neuron/glia proportion. The *lmFit* function was used also to calculate the interaction between sex and age, correcting for neuron/glia proportion, and between AD and sex, correcting for age and neuron/glia proportion. Effect sizes and standard errors were extracted from *limma* output. For each brain region, the results obtained in the different datasets were combined by inverse variance-weighted fixed-effects meta-analysis using METAL software (Willer et al., 2010). Finally, the *p*-values resulting from each meta-analysis were adjusted for multiple comparisons using the Benjamini-Hochberg (BH) procedure. Only probes with a BH-corrected *p*-value <0.01 and with concordant effect sizes between all the datasets included in each meta-analysis were retained as significant.

To identify DMPs specific for a certain brain region, we first selected the probes having a BH-corrected *p*-value <0.01 in one region and a BH-corrected *p*-value >0.01 in all the other regions; we further refined these lists by selecting the probes having large effect sizes (<5th percentile or >95th percentile) in the brain region under investigation and small absolute effect sizes (<0.1 for sex analysis; <0.001 for age analysis; <0.1 for AD analysis) in all the other regions.

### Enrichment and Gene Ontology Analysis

Enrichment of genomic regions (islands, N- and S-shores and shelves, open sea regions) was calculated using Fisher exact test, as implemented in the *fisher.test* function from the *stats* R package (*p*-value <0.05). Enrichment of Gene Ontology (GO) terms was calculated using the *methylgometh* function implemented in the *methylGSA* R package (Ren and Kuan, 2019), and redundant significant GO terms (BH-corrected *p*-value <0.01) were removed by REViGO software (Supek et al., 2011).

## Results

The selection criteria of publicly available DNAm datasets of healthy and AD human brains are described in Materials and Methods section, and the datasets included in the meta-analysis are reported in Tables 1, 2. An overview of the study design is reported in Supplementary Figure 1.

### DNA Methylation Differences Across Sex

To identify sex-dependent differentially methylated positions (sDMPs) we performed an epigenome wide association study (EWAS) in each dataset and brain region separately, considering healthy subjects and correcting for age and estimated neuron/glia proportion (Section Materials and Methods). We then conducted a meta-analysis within each brain region.

**Figure 1:**
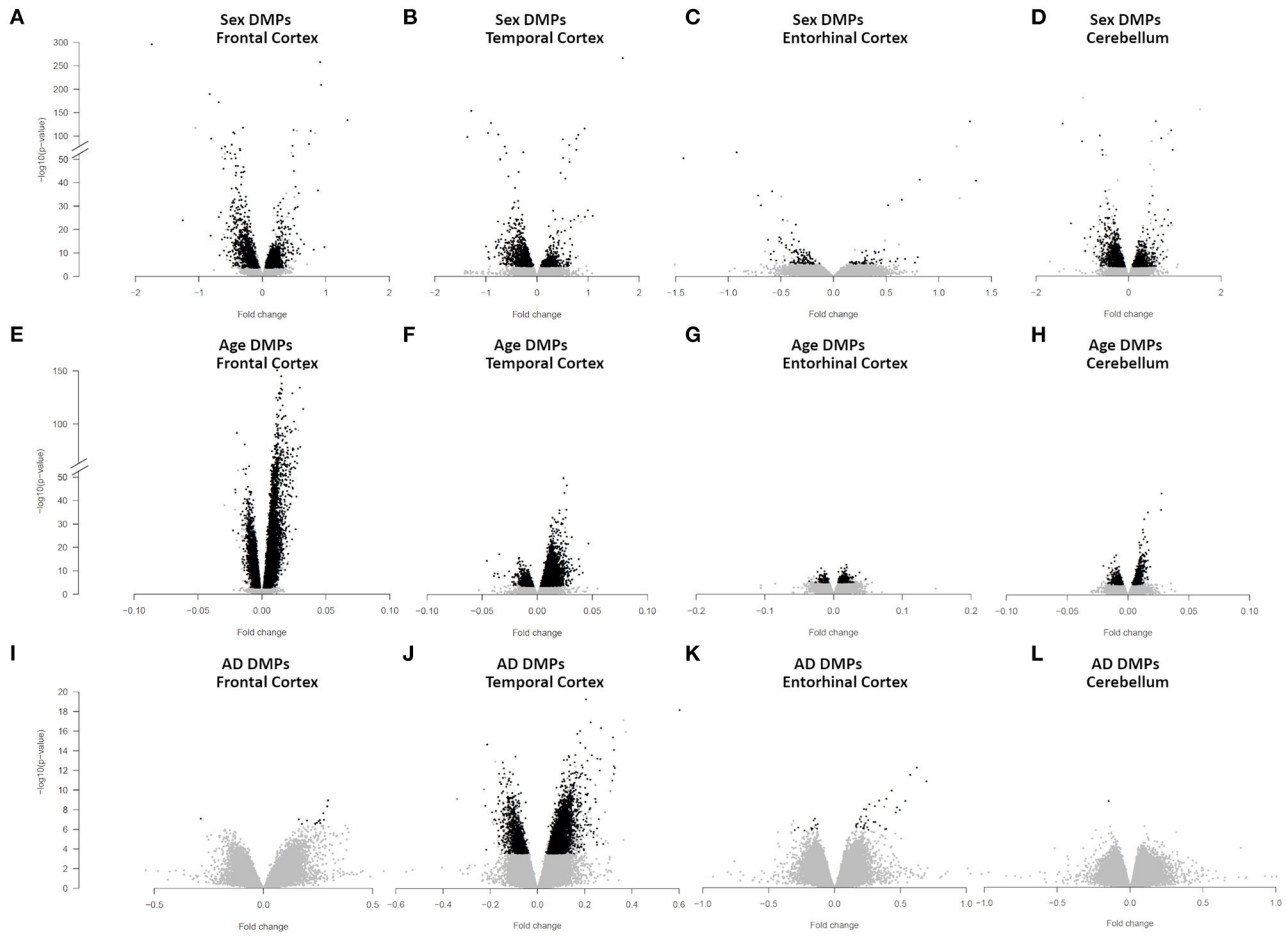
Sex-, age-, and AD-associated epigenetic changes in the four brain regions. Volcano plots of -log10(*P*-value) against effect sizes, resulting from the meta-analysis of: (i) sex-associated DMPs in FC **(A)**, TC **(B)**, ERC **(C)**, and CRB **(D)**; (ii) age-associated DMPs in FC **(E)**, TC **(F)**, ERC **(G)**, and CRB **(H)**; (iii) AD-associated DMPs in FC **(I)**, TC **(J)**, ERC **(K)**, and CRB **(L)**. Significant probes (BH-corrected *p*-value <0.01) are colored in black.

We identified 4,860 sDMPs in FC, 1,985 sDMPs in TC, 159 sDMPs in ERC, and 2,322 sDMPs in CRB (Figures 1A–D, Supplementary Figure 2, and Supplementary File 1).

In FC, sDMPs were mainly hypermethylated in males compared to females (73% of hypermethylated probes) while the opposite was true for TC, ERC, and CRB (38, 33, and 36% of hypermethylated probes in TC, ERC, and CRB, respectively). When analyzing the genomic context of the sDMPs, we found that CpG islands were enriched in sDMPs in all the four brain regions, and that CpG island shores showed a similar trend (Supplementary File 2). Also the distribution of sDMPs across chromosomes was not random, with a trend toward enrichment in chromosome 19 in all the four brain regions. The enrichment analysis of GO terms did not reveal significant results except for FC, where the “homophilic cell adhesion via plasma membrane adhesion molecules” ontology was found (Supplementary File 2).

**Figure 2:**
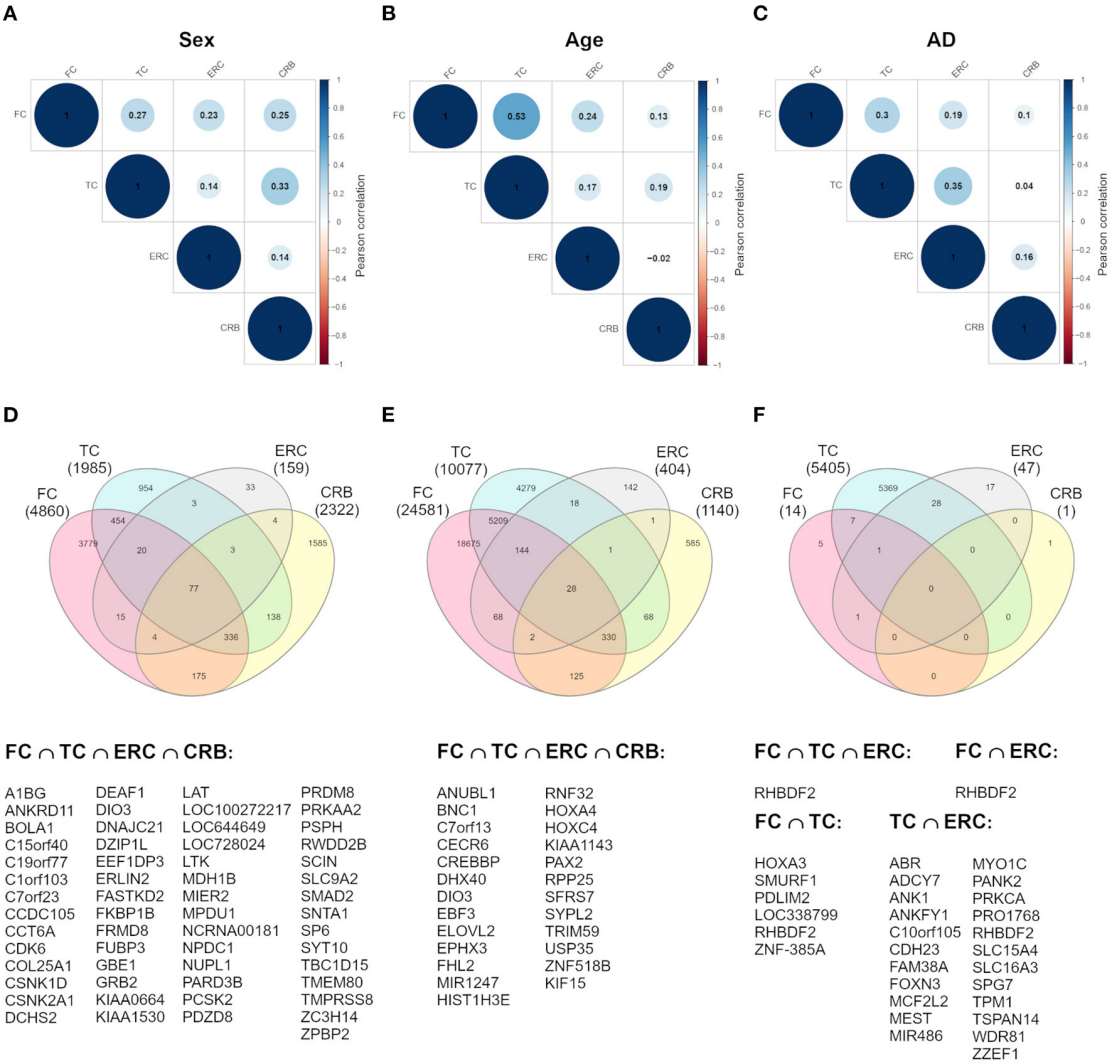
Cross-region analysis of sex-, age-, and AD-associated probes. **(A–C)** The correlation matrix plots show the magnitude of correlation among probes' effect sizes in the four brain regions, considering the results of the meta-analysis on sex- **(A)**, age- **(B)**, and AD- **(C)** associated probes. Positive and negative correlation values are indicated in blue and red, respectively. **(D–F)** The Venn diagrams display the number of significant DMPs shared between the four brain regions, considering sDMPs **(D)**, aDMPs **(E)**, and AD-DMPs **(F)**. The genes in which the most shared probes map are reported below each diagram.

To investigate whether sex-dependent DNAm changes were consistent across brain regions, we evaluated the correlation of effect size values between FC, TC, ERC, and CRB (Figure 2A). The four brain regions were positively correlated each other. We next intersected the 4 sDMPs lists, identifying 77 common probes mapping in 57 genes (Figure 2D, Table 3, and Supplementary File 1).

**Table 3:** sDMPs resulting from cross-region analysis.

**Probe**	**Chr**	**MAPINFO**	**Relation**	**Gene**	**Effect size direction**	**Yusipov et al**.	**Cited in previous studies in relation to sex**
cg00097357	12	33591336	N_Shore	SYT10	-	X	
cg00655923	7	64895418			+	X	
cg00760935	4	15541	Island	DCHS2	-	X	
cg01063965	11	695461	Island	TMEM80, DEAF1	-		Involved in sex-dependent anxiety and depression (Luckhart et al., 2016; Philippe et al., 2018)
cg01181499	2	74739419	N_Shore		-	X	
cg01906879	3	81811016	S_Shore	GBE1	-	X	
cg02093808	4	77342011	Island		-	X	
cg02297043	1	75590912	Island		-	X	
cg02530860	8	14436	Island		+	X	
cg03168896	3	44036098	N_Shore		-	X	
cg03405128	4	77341841	N_Shore		-	X	
cg03687700	2	24271844	N_Shore	FKBP1B	-	X	
cg03894796	8	13783	Island		+	X	
cg04946709	16	59789030	Island	LOC644649	+	X	
cg05020125	8	37605552		LOC728024, ERLIN2	-	X	
cg05056638	8	24800824	S_Shore		-	X	
cg05100634	18	45457604	Island	SMAD2	-		Sex-differences in extracellular matrix production (Wu et al., 2015; Dworatzek et al., 2016; Altinbas et al., 2019; Avouac et al., 2020)
cg05468028	21	30391383	Island	RWDD2B	-	X	
cg05849319	11	65172370	Island	FRMD8	+	X	
cg06666376	19	3480596	N_Shore	C19orf77	+	X	
cg06710937	13	23489940	Island		-	X	
cg07462804	4	81105375	Island	PRDM8	-	X	
cg07645761	16	2892518	N_Shore	TMPRSS8	+	X	
cg07953307	16	29000920		LAT	+	X	
cg08541880	3	13783	Island	DZIP1L	-	X	
cg09045105	1	149871	Island	BOLA1	-	X	
cg09725915	2	70369583	Island		-	X	
cg09971754	16	89557657	Island	ANKRD11	+	X	
cg10546176	5	34929404	Island	DNAJC21	-	X	
cg10749792	7	56119218	Island	PSPH, CCT6A	-	X	
cg10776186	13	25875020	Island	NUPL1	-	X	Sex-dependent differentially methylated gene (McCarthy et al., 2014)
cg11065518	2	20763	S_Shore	MDH1B, FASTKD2	-	X	
cg11174255	4	1513259	N_Shore		+	X	
cg11240062	8	14436	Island		+	X	
cg11565911	12	72233249	N_Shore	TBC1D15	-	X	
cg11841231	2	20554		PARD3B	+	X	
cg12356266	8	99984350	N_Shore		-	X	
cg12611527	2	15725	Island		-	X	
cg12611723	9	139940	Island	NPDC1	-	X	
cg13230424	17	45930033	S_Shore	SP6	-	X	
cg13346869	8	37605517		LOC728024, ERLIN2	-	X	
cg14030268	10	11913	Island	PDZD8	-	X	
cg14373579	9	13345	Island	LOC100272217, FUBP3	-	X	
cg15148078	19	3480561	N_Shore	C19orf77	+	X	
cg15817705	1	20940	S_Shore		+	X	
cg16021159	1	57142074		PRKAA2	+	X	
cg16374663	15	41805031	Island	LTK	-	X	
cg17561891	7	86849173	Island	C7orf23	-	X	
cg17743279	7	92463268	Island	CDK6	-	X	
cg17887478	17	7486551	Island	MPDU1	-	X	
cg18001427	21	30391784	S_Shore	RWDD2B	-	X	
cg18721420	19	15121913	Island	CCDC105	-	X	
cg19292062	20	524344	Island	CSNK2A1	-	X	
cg19311244	4	77341912	N_Shore		-	X	
cg19864758	20	17206720	Island	PCSK2	-	X	
cg20050113	2	103236861	S_Shore	SLC9A2	-	X	Sex-dependent differentially methylated gene (McCarthy et al., 2014)
cg20432211	4	77342104	Island		-	X	
cg22105158	19	3480672	N_Shore	C19orf77	+	X	
cg22266749	4	110223	Island	COL25A1	+	X	
cg22345911	17	80231263	Island	CSNK1D	-	X	
cg22794378	14	89029563	Island	ZC3H14	-		
cg22799420	14	102028994	Island	DIO3	-	X	Sex-dependent regulation and functions (Sittig et al., 2011; Kim et al., 2019; Stohn et al., 2019; Stone et al., 2019)
cg22889142	19	58862398	Island	NCRNA00181, A1BG	-	X	Female-specific gene expression in liver (Gardmo and Mode, 2006; Conforto et al., 2012)
cg23001456	17	2615074	Island	KIAA0664	-	X	
cg23719534	15	10109	Island		-		
cg23880736	4	582172	Island		+	X	
cg24016844	1	11150	Island	C1orf103	+	X	
cg24126849	4	581937	N_Shore		+	X	
cg24158363	17	73401717	Island	GRB2	-	X	
cg24717799	15	83680832	S_Shore	C15orf40	-	X	
cg24990494	13	32520050		EEF1DP3	+		
cg25584814	19	345306	Island	MIER2	-	X	
cg25726513	4	1340596	Island	KIAA1530	-	X	
cg26172013	20	32031452	Island	SNTA1	-	X	
cg26516287	7	12629275		SCIN	-	X	
cg26612727	17	38024636	Island	ZPBP2	-	X	Sex-dependent DNA methylation (Ho et al., 2018)
cg27645294	17	21795257			-	X	

All these probes showed concordant sex-dependent DNAm profiles in the four brain regions and most of them (73%) were hypomethylated in males. Furthermore, 93% of them were previously described to have sex-dependent DNAm also in whole blood (Yusipov et al., 2020).

**Figure 3:**
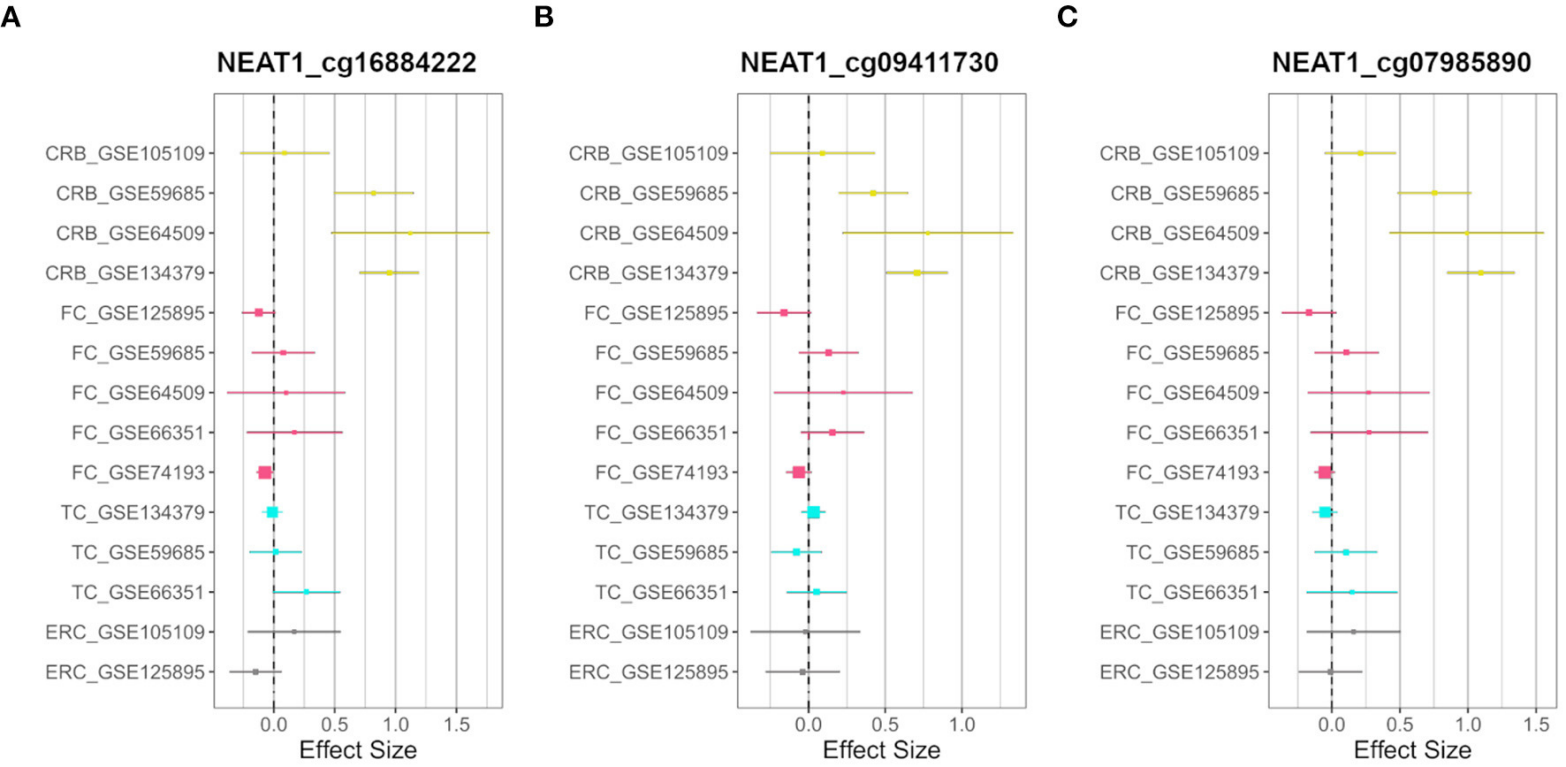
CRB-specific sex-associated DNAm of *NEAT1* gene. Forest plots of three CRB-specific sDMPs mapping in *NEAT1*gene: **(A)** cg16884222, **(B)** cg09411730, **(C)** cg07985890. For each probe, effect sizes from the datasets used for our meta-analysis are reported, dividing them according to the four brain regions (CRB, yellow; FC, magenta; TC, cyan; ERC, gray).

On the other hand, we searched for probes having sex-related DNAm differences only in one brain region (region-specific sDMPs; Section Materials and Methods). We found 2, 4, 0, and 37 region-specific sDMPs in FC, TC, ERC, and CRB, respectively (Supplementary File 1). Interestingly five sDMPs specific for CRB mapped in Nuclear Enriched Abundant Transcript 1 (NEAT1) gene (Figure 3).

### DNA Methylation Changes Across Age

To identify age-dependent differentially methylated positions (aDMPs) we performed an EWAS in each dataset and brain region separately, considering healthy subjects and correcting for sex and estimated neuron/glia proportion (Section Materials and Methods). We then conducted a meta-analysis within each brain region.

We identified 24,581, 10,077, 404, and 1,140 aDMPs in FC, TC, ERC, and CRB, respectively (Figures 1E–H, Supplementary Figure 3E, and Supplementary File 3). In all brain regions, most of the aDMPs underwent hypermethylation with age (76, 88, 58, and 62% of hypermethylated aDMPs in FC, TC, ERC, and CRB, respectively). The genomic context of aDMPs was not consistent across the four brain regions, except for a significant under-representation in “open sea” regions (Supplementary File 4). Similarly, aDMPs were differently scattered across chromosomes in FC, TC, ERC, and CRB. GO enrichment analysis revealed several pathways involved in morphogenesis and developmental processes, with “pattern specification process” and “regionalization” common to FC, TC, and ERC (Supplementary File 4).

The analysis of correlation between the effect sizes revealed that age-associated changes were more similar between FC and TC compared to the other regions (Figure 2B). The intersection of the aDMPs from the 4 brain regions highlighted 28 common probes, all concordantly undergoing hypermethylation with age and mapping in 25 genes (Figure 2E and Table 4). Again, 93% of these probes were reported as age-associated also in while blood (Yusipov et al., 2020).

**Table 4:** aDMPs resulting from cross-region analysis.

**Probe**	**Chr**	**MAPINFO**	**Relation**	**Gene**	**Effect size direction**	**Yusipov et al**.	**Cited in previous studies in relation to age**
cg00292135	7	156433068	Island	C7orf13, RNF32	+	X	
cg04090392	15	83952774	Island	BNC1	+	X	Testicular premature aging (Li J. Y. et al., 2020)
cg06639320	2	106015739	Island	FHL2	+	X	Epigenetic changes in aging (Garagnani et al., 2012; Steegenga et al., 2014; Bacos et al., 2016; Kananen et al., 2016; Bacalini et al., 2017; Spólnicka et al., 2018b)
cg06942814	7	27170819	S_Shore	HOXA4	+	X	Epigenetic dysregulation in progeroid syndrome (Maierhofer et al., 2019)
cg07303143	3	44803452	Island	KIAA1143, KIF15	+	X	
cg07525420	10	131761181	Island	EBF3	+	X	
cg07922606	6	26225389	Island	HIST1H3E	+		Regulation of age-dependent gene expression (Crossland et al., 2017)
cg11614451	3	160167729	Island	TRIM59	+		Epigenetic changes in aging (Spólnicka et al., 2018a,b; Wezyk et al., 2018)
cg12373771	22	17601381	Island	CECR6	+	X	
cg13327545	10	22623548	Island		+	X	
cg14020846	14	103674272	Island		+	X	
cg14556683	19	15342982	Island	EPHX3	+	X	
cg15243034	11	77907656	Island	USP35	+	X	
cg15341124	14	102027734	Island	DIO3, MIR1247	+	X	Age-dependent expression (McCann and Ames, 2011; Kim et al., 2014; White et al., 2015; Mikovic et al., 2018; Wang et al., 2020)
cg15611336	15	75248496	Island	RPP25	+	X	
cg16295725,	4	10459219	Island	ZNF518B	+	X	Pancreatic aging (Bacos et al., 2016; Bou Sleiman et al., 2020)
cg23995914		10459228					
cg16867657	6	11044877	Island	ELOVL2	+	X	Epigenetic changes in aging (Garagnani et al., 2012; Steegenga et al., 2014; Rönn et al., 2015; Bacalini et al., 2017; Slieker et al., 2018; Spólnicka et al., 2018b; Sturm et al., 2019; Chao and Skowronska-Krawczyk, 2020; Chen et al., 2020; Li X. et al., 2020)
cg16969368	17	57642752	Island	DHX40	+	X	
cg18008766	2	38978896	S_Shore	SFRS7	+	X	
cg18240400	10	46168597	Island	ANUBL1	+	X	
cg18473521	12	54448265	S_Shore	HOXC4	+	X	
cg19399220	19	10527588	Island		+	X	
cg20591472	1	110008990	Island	SYPL2	+	X	
cg24079702	2	106015771	Island	FHL2	+	X	Epigenetic changes in aging (Garagnani et al., 2012; Steegenga et al., 2014; Bacos et al., 2016; Kananen et al., 2016; Bacalini et al., 2017; Spólnicka et al., 2018b)
cg24567591	16	3931229	Island	CREBBP	+	X	Memory performance in elderly (Barral et al., 2014)
cg24903144	10	102509268	Island	PAX2	+	X	Retina aging (Mansour et al., 2008)
cg26092675	6	26225258	N_Shore	HIST1H3E	+	X	Regulation of age-dependent gene expression (Crossland et al., 2017)

The opposite analysis, i.e., the identification of region-specific aDMPs (section Materials and Methods), identified only one probe specific for FC (cg01725130), that maps in the body of Ras And Rab Interactor 3 (RIN3) gene (Supplementary File 2).

### The Relation Between Age and Sex in Brain DNA Methylation

We then aimed at studying how sex-specific brain DNAm is modulated during aging.

**Figure 4:**
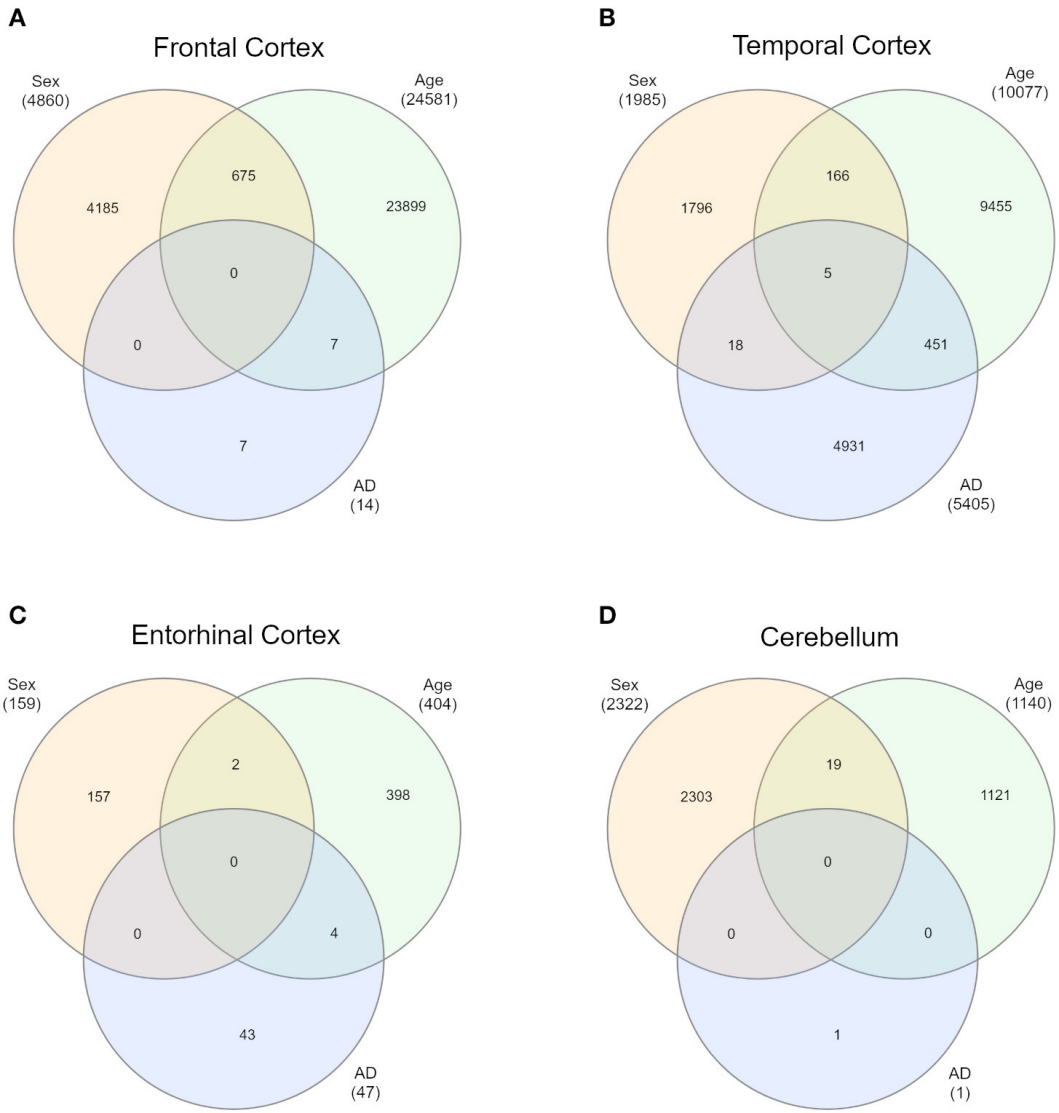
Intersections of sex-, age-, and AD-associated probes in each of the four brain regions. Venn diagrams depict the intersection between sDMPs, aDMPs, and AD-DMPs in FC **(A)**, TC **(B)**, ERC **(C)**, and CRB **(D)**.

First of all, we intersected sDMPs and aDMPs lists. In FC, we found 675 probes that change with sex and with age (s&aDMPs), corresponding to about 13% of all sDMPs identified. In TC s&aDMPs were 171, corresponding to 8.5% of sDMPs. In ERC we found only 2 s&aDMPs, while in CRB s&aDMPs were 19, corresponding to 4% of sDMPs (Figure 4 and Supplementary Files 1, 3). In all the four regions, the proportion of sDMPs changing with age (i.e., the proportion of s&aDMPs) was higher than expected (Fisher's Exact Test *p*-value <0.05; odds ratio of 2.6, 3.8, 13.0, and 3.0 in FC, TC, ERC, and CRB, respectively). In FC, TC, and CRB, most of the s&aDMPs were probes having higher DNAm levels in males respect to females and undergoing hypermethylation during aging. GO analysis revealed only one ontology enriched in FC (“homophilic cell adhesion via plasma membrane adhesion molecules”).

The previous analysis identifies CpG probes whose DNAm varies according to both sex and age, but is not informative about possible differences in aging trajectories between males and females. To fulfill this point, we performed an age-by-sex interaction analysis in each dataset (Section Materials and Methods) and meta-analyzed the results for the four brain regions. Only 4, 4, 2, and 2 probes showed a significant age-by-sex interaction in FC, TC, ERC, and CRB, respectively (Supplementary File 5).

### Brain DNA Methylation Changes Across AD

Then, we focused on brain DNAm datasets including late-onset AD patients and age-matched non-demented controls.

To identify differentially methylated positions associated with AD (AD-DMPs) we performed an EWAS in each dataset and brain region separately, correcting for age, sex, and estimated neuron/glia proportion (section Materials and Methods). We then conducted a meta-analysis within each brain region.

We identified 14 AD-DMPs in FC, 5405 in TC, 47 in ERC, and only 1 in CRB (Figures 1I–L, Supplementary Figure 4, and Supplementary File 6). In all brain regions most of AD-DMPs were hypermethylated in AD compared to controls (93, 80, 76, and 100% in FC, TC, ERC, and CRB, respectively). While in TC AD-DMPs were significantly under-represented in CpG islands and enriched in the other genomic contexts, a significant enrichment in CpG islands was found for AD-DMPs identified in FC (Supplementary File 7). GO analysis returned significant results only in TC, where pathways related to synapse organization and function were found (Supplementary File 7).

Correlation analysis of effect sizes between the four brain regions highlighted a distinctive pattern in CRB respect to FC, TC, and ERC, while the correlation was higher between TC and ERC (Figure 2C). Accordingly the intersection between AD-DMPs in the 4 brain regions did not return common probes, while 29 probes (mapping in 23 genes) and 8 probes (mapping in 6 genes) were identified by intersecting TC and ERC or FC and TC, respectively (Figure 2F and Table 5). The probe cg12163800, mapping in Rhomboid 5 Homolog 2 (RHBDF2) gene, was significantly hypermethylated in FC, TC, and ERC from AD patients. A comparison with AD-associated probes retrieved in a recent meta-analysis (Smith R. G. et al., 2020) is also reported in Table 5.

**Table 5:** AD-DMPs resulting from cross-region analysis.

**Intersection**	**Probe**	**Chr**	**MAPINFO**	**Relation**	**Gene**	**Effect size direction**	**Smith et al**.	**Cited in previous studies in relation to AD**
**TC ∩ ERC**	cg00851830	14	100201016	N_Shelf		+		
	cg03169557	16	89598950		SPG7	+	X	Retinal nerve fiber layer loss; AD DMP (Wiethoff et al., 2012; Li Q. S. et al., 2020)
	cg03183618	2	134964228			+		
	cg04658038	17	64800166		PRKCA	+		Synaptic degeneration (Wang et al., 2010; Alfonso et al., 2016; Maphis et al., 2017)
	cg05066959 cg11823178	8	41519308 41519399		ANK1, MIR486	+	X	Epigenetic changes in AD; involved in memory loss (De Jager et al., 2014; Lord and Cruchaga, 2014; Lunnon et al., 2014; Chi et al., 2016; Mastroeni et al., 2017; Gasparoni et al., 2018; Higham et al., 2019; Blanco-Luquin et al., 2020; Li Q. S. et al., 2020)
	cg05397697	14	90042217		PRO1768, FOXN3	+		
	cg05417607	17	1373605	N_Shore	MYO1C	+	X	
	cg05810363,	17	74475270	Island	RHBDF2	+	X	Epigenetic changes in AD (De Jager et al., 2014; Lord and Cruchaga, 2014; Zou et al., 2019; Li Q. S. et al., 2020)
	cg12163800,		74475355					
	cg12309456		74475402					
	cg06653632	12	129281444	S_Shore	SLC15A4	+		
	cg06753513	17	3977385		ZZEF1	+		
	cg07012687	17	80195180	Island	SLC16A3	+		
	cg07571519	10	73472315		C10orf105, CDH23	+		Expression and epigenetic changes in AD (De Jager et al., 2014; Lord and Cruchaga, 2014; Humphries et al., 2015; Hu et al., 2018)
	cg09123026	17	74480528		RHBDF2	+		Epigenetic changes in AD (De Jager et al., 2014; Lord and Cruchaga, 2014; Zou et al., 2019; Li Q. S. et al., 2020)
	cg13851211	16	50321678		ADCY7	+		
	cg14025831	20	3873404	S_Shelf	PANK2	+		
	cg14761246	3	182968758	N_Shelf	MCF2L2	+		
	cg14798745	4	184315677	N_Shelf		+		
	cg18102633	19	17487776	N_Shore	PLVAP	+		
	cg18456331	10	77188318	N_Shelf		+		
	cg18923906	10	82225771		TSPAN14	+		
	cg20148994	7	130125585	N_Shore	MEST	+		
	cg21221455	15	63342288	S_Shore	TPM1	+		
	cg22090150	17	4098227		ANKFY1	+	X	
	cg22656126	17	1637206	Island	WDR81	+		
	cg25018458	17	980014	N_Shore	ABR	+	X	Hearing loss (Irimajiri et al., 2005; Oh et al., 2010; O'Leary et al., 2017; Hacohen-Kleiman et al., 2019; Liu et al., 2020)
	cg27630153	16	88845038	Island	FAM38A	+		
**FC ∩ TC ∩ ERC**	cg12163800	17	74475355	Island	RHBDF2	+	X	Epigenetic changes in AD (De Jager et al., 2014; Lord and Cruchaga, 2014; Zou et al., 2019; Li Q. S. et al., 2020)
**FC ∩ TC**	cg01463828	8	22446721		PDLIM2	+	X	
	cg02317313	12	122	Island	LOC338799	+	X	
	cg04874795	16	86477638			-	X	
	cg07061298	7	27153847	N_Shore	HOXA3	+	X	Epigenetic changes in AD (Gasparoni et al., 2018; Hernández et al., 2018; Smith et al., 2018; Li Q. S. et al., 2020)
	cg12163800	17	74475355	Island	RHBDF2	+	X	Epigenetic changes in AD (De Jager et al., 2014; Lord and Cruchaga, 2014; Zou et al., 2019; Li Q. S. et al., 2020)
	cg22962123	7	27153605	Island	HOXA3	+	X	
	cg26022064	7	98739782	N_Shore	SMURF1	+	X	Neural necroptosis and Hirano bodies (Makioka et al., 2014; Shao et al., 2018)
	cg26199857	12	54764265	Island	ZNF385A	+		
**FC ∩ ERC**	cg12163800	17	74475355	Island	RHBDF2	+	X	
	cg13076843		74475294					

### Confirmation of Sex- and Age-Associated DNAm Changes in AD Subjects

We investigated whether the sDMPs and aDMPs identified above in the different brain regions from healthy controls were confirmed also in AD patients. To this aim, we evaluated their association with sex (correcting for age and estimated neuron/glia proportion) or with age (correcting for sex and estimated neuron/glia proportion) considering DNAm data from AD samples, and performed a meta-analysis in each brain region. The effect sizes obtained in AD were highly correlated with those previously obtained in healthy controls (Supplementary Figure 5). This correlation was slightly lower for aDMPs, which is expected considering that in most datasets the age range tends to be narrower for AD samples compared to healthy controls. Interestingly, also in AD samples we found an enrichment of sDMPs on chromosome 19 (data not shown). Collectively, these results indicate that sex- and age-dependent DNAm patterns are largely reproduced in AD samples.

### The Relationship Between Sex- and Age-Associated DNAm Changes and AD Epigenetic Remodeling

We explored whether AD-associated DNAm changes were related to sex- and age-specific brain DNAm patterns occurring in physiological conditions, identified in the analyses described above.

In each brain region, we intersected the AD-DMPs and sDMPs in order to identify AD&sDMPs, i.e., probes that have basal differential DNAm between the two sexes and are also affected by AD. The intersection did not result in any probe for all the regions except that for TC, where we found 23 AD&sDMPs, mapping in 16 genes and corresponding to only 0.4% of AD-DMPs in TC (Fisher's Exact Test *p*-value >0.05; Figure 4 and Supplementary Files 1, 6). Moreover, AD-by-sex interaction analysis yielded no significant probes in any region.

To further explore the epigenetic relationship between sex and AD, we extended our analysis to probes located on sex chromosomes and focused on AD datasets in which their DNAm values were available (Section Materials and Methods). For each dataset, we considered males and females separately, we repeated the EWAS for AD-associated DNAm and we performed the meta-analysis within each brain region. We then searched for significant AD-DMPs located on the X or Y chromosomes. This analysis returned only few probes: four X-linked DMPs were found in TC when males with and without AD were compared, while one X-linked probe was found in male ERC (Supplementary File 6).

**Figure 5:**
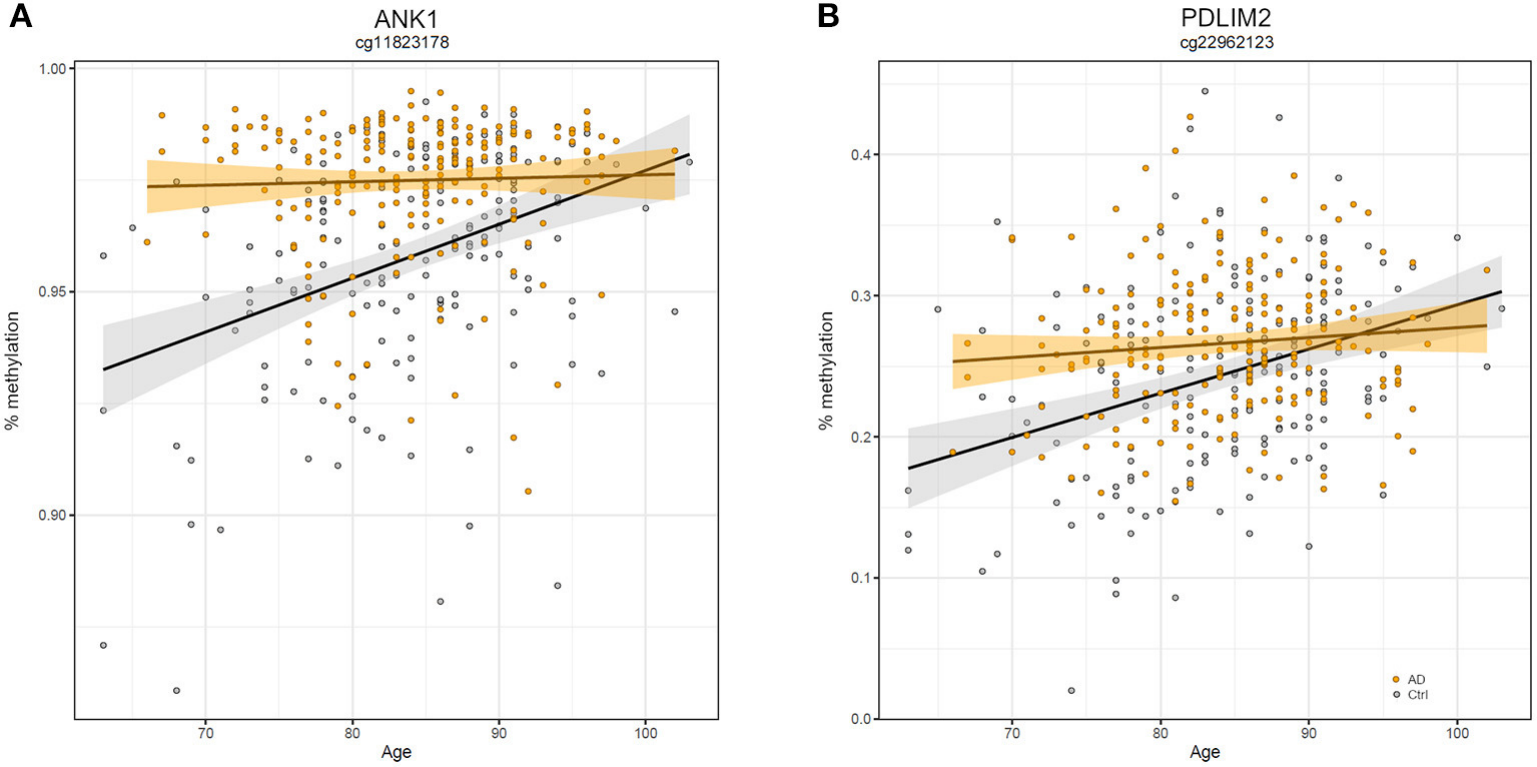
Scatter plots of *ANK1* and *PDLIM2* DNAm according to age and disease. Scatter plots of methylation values of cg11823178 within *ANK1* **(A)** and of cg22962123 within *PDLIM2* **(B)** in TC from GSE134379 dataset. Healthy subjects are colored in gray while AD patients are in orange. Regression lines and confidence intervals within each group are reported.

Similarly, we explored whether AD-DMPs occur in probes whose DNAm varies during physiological aging (AD&aDMPs). The intersection between AD-DMPs and aDMPs highlighted 7, 456, 4, and 0 probes in FC, TC, ERC, and CRB, respectively (Figure 4). The proportion of AD&aDMPs was higher than expected by chance in FC, TC, and ERC (Fisher's Exact Test *p*-value <0.05; odds ratio of 15.9, 3.8, and 95 in FC, TC, and ERC, respectively). We found that 87% of AD&aDMPs in TC are concordant for the effect size sign between aDMPs and AD-DMPs, while this percentage reached 100% in FC and ERC. Notably, the four AD&aDMPs found in ERC (cg11823178, cg03169557, cg25018458, and cg22090150) were also found in TC (Table 6). Also the intersection between AD&aDMPs in TC and FC returned four common probes (cg01463828, cg04874795, cg22962123, and cg07061298; Table 6). Figure 5 reports DNAm values of cg11823178 (ANK1) and cg22962123 (PDLIM2) in TC from GSE134379 dataset as an example of CpG sites displaying a positive association of DNAm with age and hypermethylated in AD.

**Table 6:** AD&aDMPs resulting from cross-region intersections.

**Intersection**	**Probe**	**Chr**	**MAPINFO**	**Relation**	**Gene**	**Effect size direction**	**Smith et al**.	**Yusipov et al**.
								**(Bonf. Corrected aDMPs)**
**ERC ∩ TC**	cg11823178	8	41519399		ANK1	+	X	X
	cg03169557	16	89598950		SPG7	+	X	
	cg25018458	17	980014	N_Shore	ABR	+	X	
	cg22090150	17	4098227		ANKFY1	+	X	
**FC ∩ TC**	cg22962123	7	27153605	Island	HOXA3	+	X	X
	cg07061298	7	27153847	N_Shore	HOXA3	+	X	
	cg04874795	16	86477638			-	X	X
	cg01463828	8	22446721		PDLIM2	+	X	

Finally, it is worth to note that TC is the only brain region in which we found probes at the intersection between aDMPs, sDMPs, and AD-DMPs (AD&a&sDMPs; Figure 4B). The five probes (cg20225999, cg03951603, cg08820801, cg22263793, cg10828284; Table 7) were all hypermethylated in males and with aging; three of them (cg20225999, cg08820801, cg10828284) were further hypermethylated in AD.

**Table 7:** Probes resulting from the intersection between aDMPs, sDMPs, and AD-DMPs in TC.

**Probe**	**Chr**	**MAPINFO**	**Relation**	**Gene**	**Effect size direction**	**Smith et al**.	**Yusipov et al**.	**Yusipov et al**.
cg20225999	2	218843435	N_Shore		+	X		X
cg03951603	15	89903565	Island		-		X	
cg08820801	19	39465821	N_Shore	FBXO17	+		X	
cg22263793	19	42501398	Island		-		X	
cg10828284	22	50528333	Island	MOV10L1	+		X	

### Contribution of 5-hydroxymethylcytosine to the Epigenetic Changes Across Sex, Age, and AD

All the analyses reported above are based on microarray data from BS converted DNA. BS treatment does not allow to distinguish between 5mC and 5hmC, another epigenetic mark which plays an important role especially in the brain (Kriaucionis and Heintz, 2009; Lunnon et al., 2016). On the contrary, the combination of BS with oxBS treatment allows discriminating the levels of 5mC, 5hmC, and 5uC in DNA (Booth et al., 2012). One of the datasets that we used in our meta-analysis for sex-, age-, and AD-associated epigenetic changes (GSE105109) includes microarray results from matched BS- and OxBS-treated ERC and CRB samples. We calculated the levels of 5mC, 5hmC, and 5uC (Section Materials and Methods) in this dataset and we analyzed them for the association with sex (in healthy subjects), with age (in healthy subjects), and with AD (comparing AD and healthy subjects). This analysis did not return any significant probe, neither in ERC nor in CRB. We then considered the lists of sDMPs, aDMPs, and AD-DMPs identified in the meta-analysis of ERC and CRB datasets, and used GSE105109 data to investigate the contribution of 5mC, 5hmC, and 5uC to the observed epigenetic changes. Supplementary Figure 6 reports the correlation between the effect size values resulting from the meta-analysis of sex, age, and AD, and the effect size values obtained in GSE105109 dataset using 5mC, 5hmC, and uC values in the association analysis. In both ERC and CRB, sDMPs and AD-DMPs showed high correlation between BS (5mC+5hmC) results and oxBS (5mC) results, while the correlation with 5hmC results was low. A similar trend was observed for aDMPs in CRB. This indicates that 5mC is the main contributor to the epigenetic changes observed for the sDMPs and the AD-DMPs in ERC and CRB, and for the aDMPs in CRB. On the contrary, for ERC aDMPs, BS-effect sizes were similarly correlated with 5mC- and 5hmC-effect sizes, indicating that both the epigenetic marks are remodeled during aging in this brain region. Furthermore, age-associated changes in 5mC and 5hmC were likely to involve different probes, as 5mC and 5hmC effect sizes were not clearly correlated.

## Discussion

Sex and age are among the major risk factors for AD. In this paper, we performed a meta-analysis of DNAm changes that are associated to sex and aging in four brain regions (FC, TC, ERC, CRB) and we evaluated whether they contribute to the epigenetic alterations that have been widely described in AD. Our main findings are discussed in the following paragraphs.

### Sex-Dependent DNAm Differences Tend to Be Shared Between Brain Regions, With Few Exceptions

To date some studies have reported DNAm sex differences in human brain, mainly focusing on frontal cortex (Xu et al., 2014; Spiers et al., 2015; Masser et al., 2017; Perzel Mandell et al., 2020) with few exceptions (Xia et al., 2019). Our meta-analysis confirms the presence of autosomic probes with differential methylation between males and females in all the brain regions. These probes preferentially map in CpG islands and shores suggesting their involvement in the regulation of sex-specific gene expression in brain (Xu et al., 2014). Surprisingly, in all the brain regions we found an enrichment of sDMPs in chromosome 19. This observation is difficult to be explained but a similar result was observed in a precedent study on sex-associated DNAm differences across childhood in whole blood (Suderman et al., 2017). Chromosome 19 has the highest content of CpG sites and genes in the genome (Grimwood et al., 2004), and seems to be involved in the process of X chromosome inactivation (Migeon et al., 2017).

Sex specific DNAm tended to be reproducible across the brain regions and 77 CpGs resulted from the cross-region intersection. Among them there are sDMPs mapping in genes that have been already associated to sex differences in brain physiology and pathology, like Par-3 Family Cell Polarity Regulator Beta (*PARD3B*) (Phillips et al., 2019), DEAF1 Transcription Factor (*DEAF1*) (Luckhart et al., 2016), and Iodothyronine Deiodinase 3 (*DIO3*) (Stohn et al., 2019) genes. Most of these 77 probes were previously reported as differentially methylated between males and females also in previous meta-analysis on blood (McCarthy et al., 2014; Yusipov et al., 2020).

In addition, we found few examples of sDMPs specific for a brain region. The most notable example is in cerebellum and maps in *NEAT1*. *NEAT1* is a ubiquitously expressed long non-coding RNA (lncRNA) involved in a plethora of neurospecific processes such as brain development and aging (An et al., 2018; Pereira Fernandes et al., 2018; Salvatori et al., 2020). Recent transcriptomic studies on human central nervous system revealed altered *NEAT1* levels in AD (Spreafico et al., 2018), PD (Simchovitz et al., 2019), and in schizophrenia (Katsel et al., 2019).

### DNAm Tends to Be Differently Remodeled During Aging According to the Brain Region

Several studies have analyzed age-associated changes in DNAm in brain, both comparing fetal vs. adult brains and analyzing methylation profiles across adulthood (Hernandez et al., 2011; Horvath et al., 2012; Numata et al., 2012; Day et al., 2013; Jaffe et al., 2016; Gasparoni et al., 2018; Price et al., 2019). Our meta-analysis shows that during aging there is an increase in methylation at specific loci, accordingly to previously published data on blood (Xiao et al., 2016; Yusipov et al., 2020) and brain (Hernandez et al., 2011). As previously reported by Hernandez et al. (2011), also our results support the involvement of brain aDMPs in GO related to developmental processes and morphogenesis. Furthermore, our meta-analysis confirms and extends the observation that the epigenome is differently remodeled during aging across brain regions (Hernandez et al., 2011). In particular we observed that age-associated DNAm patterns are similar in TC and FC, while they are distinct in ERC and CRB. CRB was previously described to undergo a peculiar epigenetic aging, which was decelerated according to Horvath's epigenetic clock (Horvath et al., 2015).

The large fraction (93%) of the 28 aDMPs emerged from our cross region analysis was found also in aging studies on blood (Yusipov et al., 2020). Among them there are probes mapping in Four And A Half LIM Domains 2 (*FHL2*) and ELOVL Fatty Acid Elongase 2 (*ELOVL2*) genes, previously reported as age-associated in a large number of studies on several tissues (Garagnani et al., 2012; Slieker et al., 2018) including sorted neuron and glia cells (Gasparoni et al., 2018). According to what discussed above and to previous results (Bacalini et al., 2017; Slieker et al., 2018) the effect size of *ELOVL2* probe cg16867657 was lower in CRB respect the other regions, but still significant in our meta-analysis. Elovl2 is an enzyme involved in the elongation of fatty acids and its functional role in aging has been recently suggested (Chao and Skowronska-Krawczyk, 2020).

### Sites With Sex-Dependent DNAm Are Similarly Modulated During Aging in Males and Females

Previous studies in mice and humans suggested that, while sex-differences in DNAm at certain CpG sites are maintained during life, other CpG sites show sexually divergent aging patterns, i.e., they have a different response to aging in males and females (Masser et al., 2017). Our meta-analysis supports the fact that sDMPs have a high propensity to be modulated during aging, as the number of probes resulting from the intersection of sDMPs and aDMPs is higher than expected in all the four brain regions. However, we found only few probes with significant age-by-sex interaction, indicating similar rather than diverging changes in DNAm in males and females aging. The discrepancy between our results and previous findings can be due to different reasons: for example, while Masser et al. considered only one dataset including frontal cortex data, here we meta-analyzed several datasets using selective criteria of concordance between all datasets from the same brain region; furthermore, we applied a filtering step that removed potentially ambiguous probes, thus reducing the potential overlap with Masser's results. Our results are more similar to what reported by two independent studies in blood (McCartney et al., 2019; Yusipov et al., 2020) that showed that only a small fraction of CpGs have significant age-by-sex interaction. Further studies on larger cohorts are needed to better describe sex-dependent DNAm patterns during brain aging.

### Epigenetic Changes in AD Are Enriched in Sites That Show Age-Dependent DNAm

A recent meta-analysis on EWAS studies identified 220 CpGs associated with AD neuropathology, shared by brain cortical cortex regions but not by CRB (Smith et al., 2019). The paper by Smith et al. included several datasets that we used also in our meta-analysis, with the exception of GSE125895 and GSE109627, while we did not have access to the ROS/MAP and RBD DNAm data. Furthermore, while Smith et al. considered the association with Braak stage, here we used the disease as a binary trait (affected/unaffected). Despite these differences, our results largely overlap with those previously reported. In particular, we did not find AD-related probes common to all the four brain regions that we investigated, with CRB DNAm less affected by the pathology. On the contrary, a subset of sites was shared between FC, TC, and ERC, and about 50% of these probes overlap with published data. These probes map within genes whose epigenetic deregulation has been largely documented in AD, including *ANK1, RHBDF2*, and *HOXA3*. On the contrary, we did not find any overlap when comparing our results on AD brain with CpG sites identified in AD patients' blood (Roubroeks et al., 2020), confirming that the pathology differently affects the two tissues as recently reported (Wei et al., 2020).

We did not find a significant overlap between AD-DMPs and sDMPs, nor significant interaction effects between sex and AD. Overall these results suggest that AD does not predominantly insist on autosomic sites with sex-specific DNAm. Similarly, when we repeated our analysis including probes on X and Y chromosomes (analyzing males and females separately) we found limited evidence of differential DNAm between AD and controls in sex chromosomes. Collectively these results suggest that no profound sex-associated DNAm remodeling occurs in AD. However, we cannot rule out that more subtle epigenetic differences exist, both on autosomes and sex chromosomes. It is possible that these differences did not emerge from our meta-analysis, due to the stringent selection criteria that we applied or to the small sample sizes when males and females were considered separately. Further studies should investigate possible epigenetic contributions to the different AD risk between the two sexes.

Conversely, our data show that in FC, TC and ERC, AD-related epigenetic modifications are significantly enriched in probes whose DNAm varies with age. Strikingly, we found a high concordance between the direction of DNAm changes (hyper or hypo-methylation) in AD&aDMPs, indicating that a subset of age-associated DNAm changes is exaggerated in AD. In TC, AD&aDMPs included probes mapping in *ANK1*, and it is worth to note that the down-regulation of Ank2 (ANK1 human ortholog gene) in *Drosophila* has been associated to memory loss, neuronal dysfunction and shortened lifespan in a recent report (Higham et al., 2019). Other interesting genes emerged from our analysis. Paraplegin (SPG7) mutation leads to shortened lifespan, environmental stress. and muscular and neuronal degeneration in *Drosophila* (Pareek et al., 2018). Mov10 Like RISC Complex RNA Helicase 1 (MOV10L1) is a putative germline-specific RNA helicase whose expression has been recently reported to be tightly correlated with brain development, aging and AD neurodegeneration (Skariah et al., 2017; Srinivasan et al., 2020).

Overall, these results support a geroscience view (Kennedy et al., 2014; Sierra, 2020) according to which AD can be considered a deviation of the physiological aging trajectories toward accelerated aging. Epigenetic age acceleration was previously reported in AD neurons, where a pronounced loss of CpH methylation was found at enhancers, similar to what observed in aging, and in bulk prefrontal cortex, where epigenetic age calculated by Horvath's clock was positively associated with neuritic plaques and amyloid load (Levine et al., 2015). It will be interesting to know whether similar results will be obtained using the recently published epigenetic clock optimized for brain tissues (Shireby et al., 2020).

### Strengths, Limitations, and Conclusions

To the best of our knowledge, this is the first report in which sex-, age-, and AD-related DNAm changes are systematically assessed using the same analytical approach. We used stringent selection criteria that enabled to select only probes with concordant DNAm changes in the different datasets. Furthermore, we considered multiple brain regions and reported similarities and differences in their epigenetic remodeling. Previous studies showed that DNAm patterns differ between brain regions and that they may play a role in brain development and functional specialization (Ladd-Acosta et al., 2007; Rizzardi et al., 2019). These “baseline” DNAm differences can mediate disease mechanisms that are specific for certain brain regions (Rizzardi et al., 2019), and can be further modified across lifespan and in response to pathological conditions. Accordingly, brain areas are differently affected during aging and/or in AD onset and progression (Peters, 2006; Coupé et al., 2019), and also other molecular layers like transcriptomics and proteomics show region-specific changes (Patel et al., 2019; Xu et al., 2019).

On the other side, our study has some limitations. The datasets that we meta-analyzed largely vary in size and age range of the assessed subjects, an important aspect for the identification of aDMPs. Moreover, our meta-analysis included data on BS-treated DNA and it was therefore not possible to distinguish 5mC from 5hmC, an epigenetic modification that contributes to both brain function and neurodegeneration (Coppieters et al., 2014; Ellison et al., 2017; Lardenoije et al., 2019; Smith et al., 2019). The analysis of the dataset in which 5mC and 5hmC were distinguishable (thanks to the simultaneous analysis of BS- and oxBS-treated DNA) suggested that the contribution of 5hmC to sDMPs and AD-DMPs tended to be small. A recent study showed that in fetal brain autosomal 5hmC levels did not differ between males and females (Spiers et al., 2017), in accordance to our results. Global changes in 5hmC have been reported to occur in AD (Chouliaras et al., 2013; Condliffe et al., 2014; Coppieters et al., 2014), while microarray-based genome wide studies identified a limited set of CpG sites whose hmC levels are associated to the disease (Lardenoije et al., 2019; Smith et al., 2019). For example, Smith et al. reported that AD-associated hypermethylation of *ANK1* detected on BS-treated DNA is not due to an increase in 5hmC levels, which on the contrary decreased in the disease (Smith et al., 2019). Similarly, in our analysis of AD in ERC most of the significant associations were due to changes in 5mC and we did not observe an evident co-variation of 5mC and 5hmC. On the contrary, when considering aDMPs in ERC we found a contribution of both 5mC and 5hmC, and the two epigenetic marks tended to involve different CpG sites. Age-associated increase in 5hmC levels has been previously reported (Chouliaras et al., 2012). Although potentially interesting, these results are based on only one dataset, and the analysis of the coordinated regulation of brain 5mC and 5hmC across sex, age, and AD deserves further studies. Another limitation of our study is that the datasets that we meta-analyzed were based on bulk brain tissues. Although all the analyses were corrected for neuron/glia proportions predicted from DNAm data, we cannot exclude that the observed sex-, age-, and AD-associated DNAm changes are at least in part driven by changes in brain cells composition that occur in physiological and pathological conditions. For example, Gasparoni et al. reported that *ANK1* deregulation in AD is specific for glial cells (Gasparoni et al., 2018), a finding further supported by gene expression studies (Mastroeni et al., 2017), and that the epigenetic profiles of neurons and glia are differently modulated during aging. Notwithstanding, our results suggest that the (cell-specific) age-associated remodeling of DNAm is not just a confounding factor for the epigenetic deregulation observed in AD, but on the contrary, it is the predisposing *milieu* in which AD pathogenetic mechanisms are established.

In conclusion, we suggest that age-associated DNAm patterns concur to the epigenetic deregulation observed in AD, providing new insights on how advanced age enables neurodegeneration.

## Data Availability Statement

The datasets presented in this study can be found in online repositories. The names of the repository/repositories and accession number(s) can be found in the article/Supplementary Material.

## Author Contributions

CPe, CPi, CS, MI, DM, RL, CF, PC, PG, and MGB contributed to the conception and design of the study. CPe, CS, IY, KK, DD, and MGB organized the datasets. CPe, CPi, CS, FR, IY, KK, AK, and MGB performed the statistical analysis. MGB, CPe, and CPi wrote the manuscript. All authors contributed to manuscript revision, and read and approved the submitted version.

## Conflict of Interest

The authors declare that the research was conducted in the absence of any commercial or financial relationships that could be construed as a potential conflict of interest.

## Supplementary Material

Supplementary Figure 1 Overview of the meta-analysis. For simplicity, the age-by-sex and AD-by-sex interaction analyses are not reported, but they were performed using the same pipeline illustrated in this scheme.

Supplementary Figure 2 Manhattan plots of sDMPs in the four brain regions. The figure displays the Manhattan plots resulting from the meta-analysis of sex-associated probes in FC **(A)**, TC **(B)**, ERC **(C)**, and CRB **(D)**. Significant sDMPs are marked with dark color. Scale change across 50 is indicated by an axis break.

Supplementary Figure 3 Manhattan plots of aDMPs in the four brain regions. The figure displays the Manhattan plots resulting from the meta-analysis of age-associated probes in FC **(A)**, TC **(B)**, ERC **(C)**, and CRB **(D)**. Significant aDMPs are marked with dark color. Scale change across 50 is indicated by an axis break.

Supplementary Figure 4 Manhattan plots of AD-DMPs in the four brain regions. The figure displays the Manhattan plots resulting from the meta-analysis of AD-associated probes in FC **(A)**, TC **(B)**, ERC **(C)**, and CRB **(D)**. Significant AD-DMPs are marked with dark color.

Supplementary Figure 5 Confirmation of sDMPs and aDMPs in AD patients. The scatter plots report, for the sDMPs and aDMPs identified in each tissue in healthy subjects, the effect sizes obtained in healthy subjects against the effect sizes resulting from the meta-analysis in AD patients. Pearson Correlation coefficient is reported in each plot.

Supplementary Figure 6 Contribution of 5hmC to the epigenetic changes across sex, age, and AD. Correlation plots of the effect sizes of sDMPs, aDMPs, and AD-DMPs identified in ERC and CRB, calculated using BS values (5mC+5hmC), oxBS values (5mC), BS-oxBS values (5hmC), and 1-BS values (5uC). Absolute correlation values are reported.

Supplementary File 1 Significant sDMPs in the four brain regions. The tables report the lists of sDMPs for each brain region (FC, TC, ERC, and CRB). Probes resulting from the analysis of cross-region and region-specific sDMPs are indicated by a cross, together with the probes that are in common with aDMPs or AD-DMPs found in the same region.

Supplementary File 2 Enrichment analysis of sDMPs. The tables report: (1) the results of Fisher's test on genomic distribution of sDMPs for each brain region, considering genomic context and chromosomal location. Significant results (*p*-value <0.05) are colored in green or red if depleted or enriched, respectively. (2) the results of GO pathway enrichment analysis, after REViGO filtering. Only the significant results (adjusted *p*-value <0.01) for each brain region are reported.

Supplementary File 3 Significant aDMPs in the four brain regions. The tables report the lists of aDMPs for each brain region (FC, TC, ERC, and CRB). Probes resulting from the analysis of cross-region and region-specific sDMPs are indicated by a cross, together with the probes that are in common with sDMPs or AD-DMPs found in the same region.

Supplementary File 4 Enrichment analysis of aDMPs. The tables report: (1) the results of Fisher's test on genomic distribution of aDMPs for each brain region, considering genomic context and chromosomal location. Significant results (*p*-value <0.05) are colored in green or red if depleted or enriched, respectively. (2) the results of GO pathway enrichment analysis, after REViGO filtering. Only the significant results (adjusted *p*-value <0.01) for each brain region are reported.

Supplementary File 5 Probes with significant age-by-sex interaction in the four brain regions. The tables report the lists of probes with significant age-by-sex interaction in each brain region (FC, TC, ERC, and CRB).

Supplementary File 6 Significant AD-DMPs in the four brain regions. The tables report the lists of AD-DMPs for each brain region (FC, TC, ERC, and CRB). Probes resulting from the analysis of cross-region and region-specific AD-DMPs are indicated by a cross, together with the probes that are in common with sDMPs or aDMPs found in the same region. The sheet “Sex Chromosomes” reports the significant AD-DMPs identified on sex chromosomes when analyzing males and females separately.

Supplementary File 7 Enrichment analysis of AD-DMPs. The tables report: (1) the results of Fisher's test on genomic distribution of AD-DMPs for each brain region, considering genomic context and chromosomal location. Significant results (*p*-value <0.05) are colored in green or red if depleted or enriched, respectively. (2) The results of GO pathway enrichment analysis, after REViGO filtering. Only the significant results (adjusted *p*-value <0.01) for each brain region are reported.

## Abbreviations

DNAm: DNA methylation
AD: Alzheimer's disease
DMPs: differentially methylated positions
EWAS: epigenome-wide association study
GO: gene ontology
sDMPs: sex-associated differentially methylated positions
aDMPs: age-associated differentially methylated positions
s&aDMPs: sex-, and age-associated differentially methylated positions
AD&aDMPs: late onset Alzheimer's disease-specific age-associated differentially methylated positions
AD&sDMPs: late onset Alzheimer's disease-specific sex-associated variably methylated positions
AD&a&sDMPs: late onset Alzheimer's disease-specific sex- and age-associated variably methylated positions
5mC: 5-methylcytosine
5hmC: 5-hydroxymethylcytosine
5uC: unmethylated cytosine
BS: bisulfite
oxBS: oxidative bisulfite.
