# Supplementary figures and images for: A Meta-Analysis of Brain DNA Methylation Across Sex, Age, and Alzheimer's Disease Points for Accelerated Epigenetic Aging in Neurodegeneration
Source: Front Aging Neurosci. 2021 Mar 11;13:639428. doi: 10.3389/fnagi.2021.639428 (PMC8006465; doi:10.3389/fnagi.2021.639428)

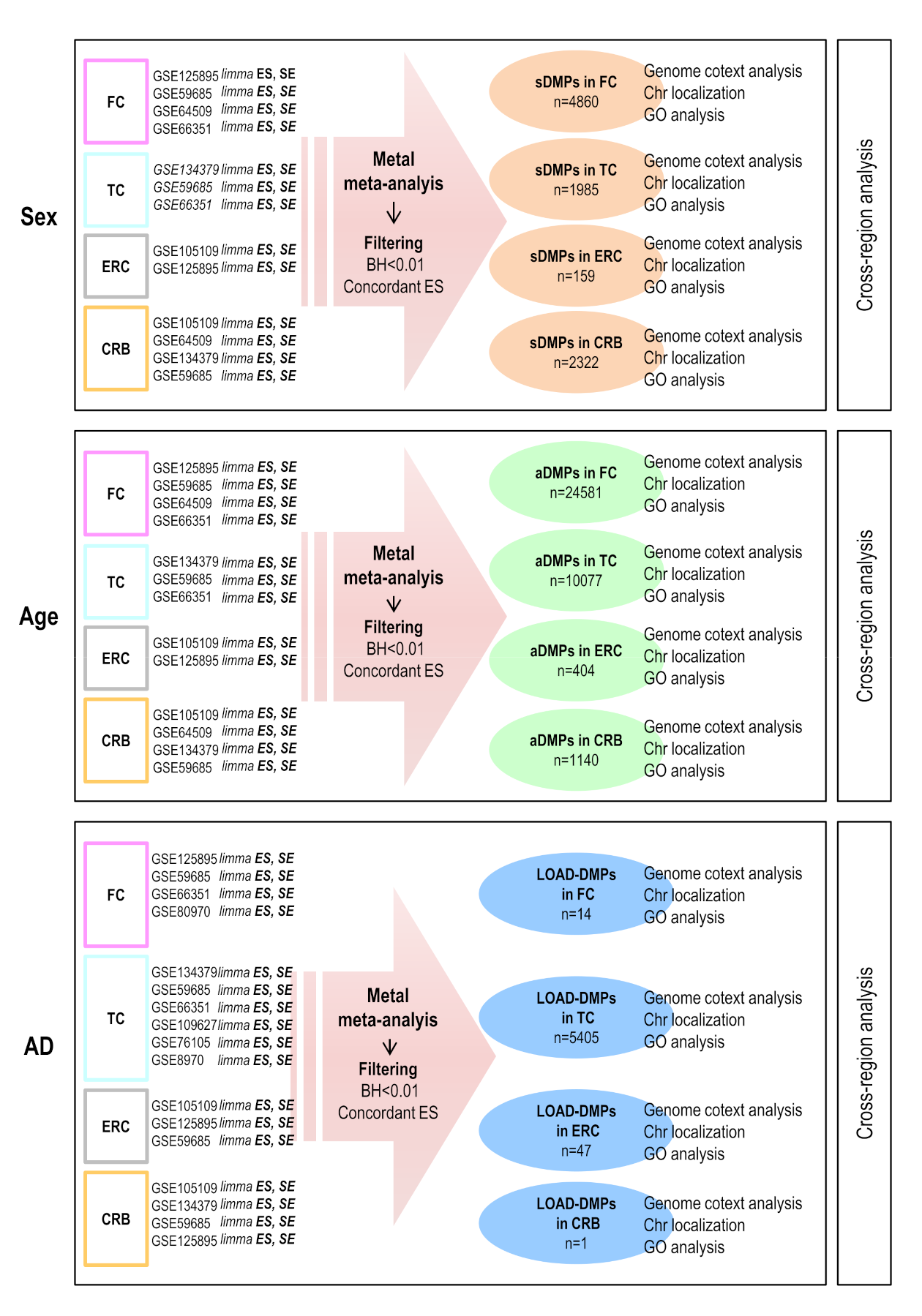

Supplement: Supplementary Figure 1 — Overview of the meta-analysis. For simplicity, the age-by-sex and AD-by-sex interaction analyses are not reported, but they were performed using the same pipeline illustrated in this scheme. [file Image_1.TIF]

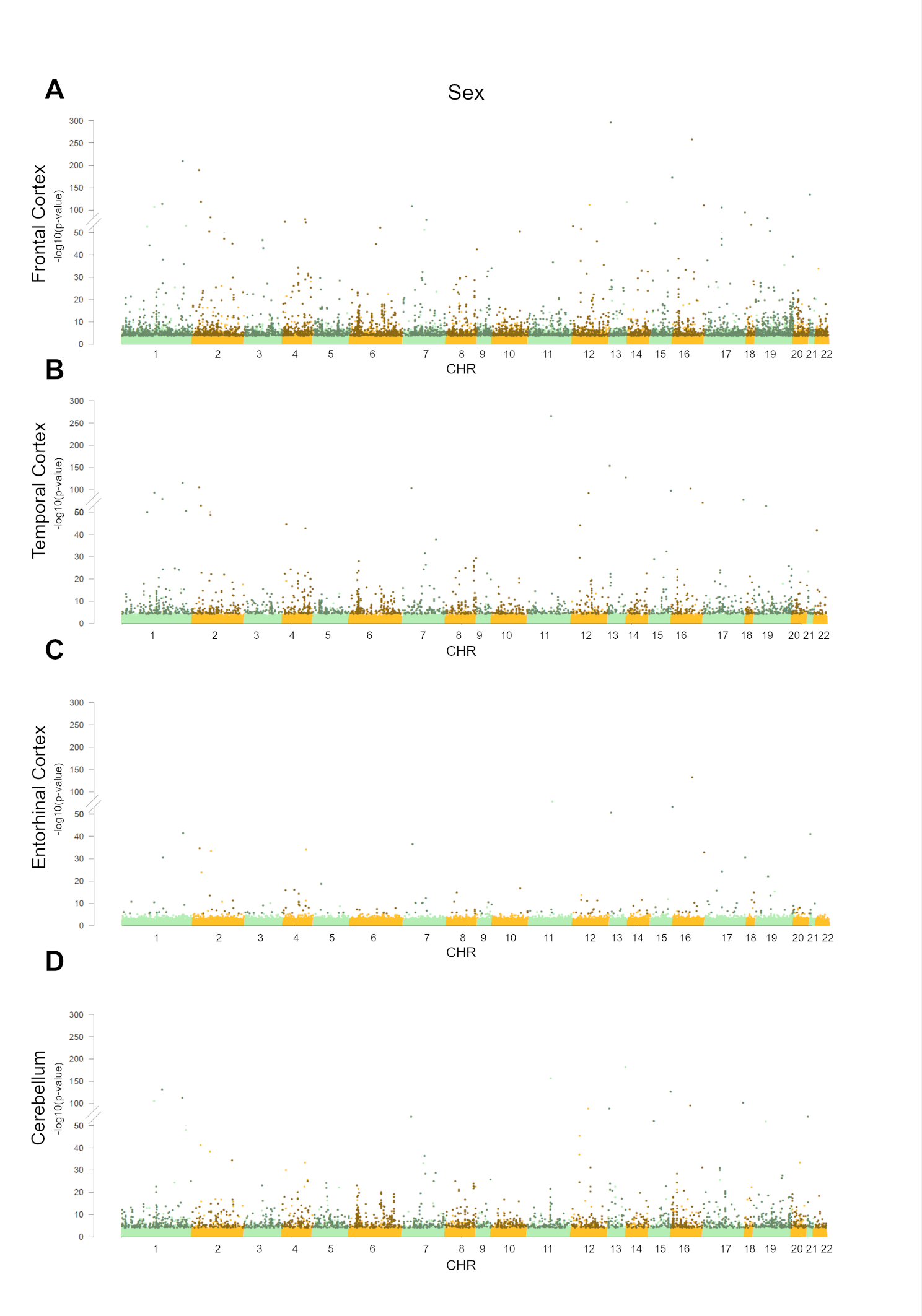

Supplement: Supplementary Figure 2 — Manhattan plots of sDMPs in the four brain regions. The figure displays the Manhattan plots resulting from the meta-analysis of sex-associated probes in FC (A), TC (B), ERC (C), and CRB (D). Significant sDMPs are marked with dark color. Scale change across 50 is indicated by an axis break. [file Image_2.TIF]

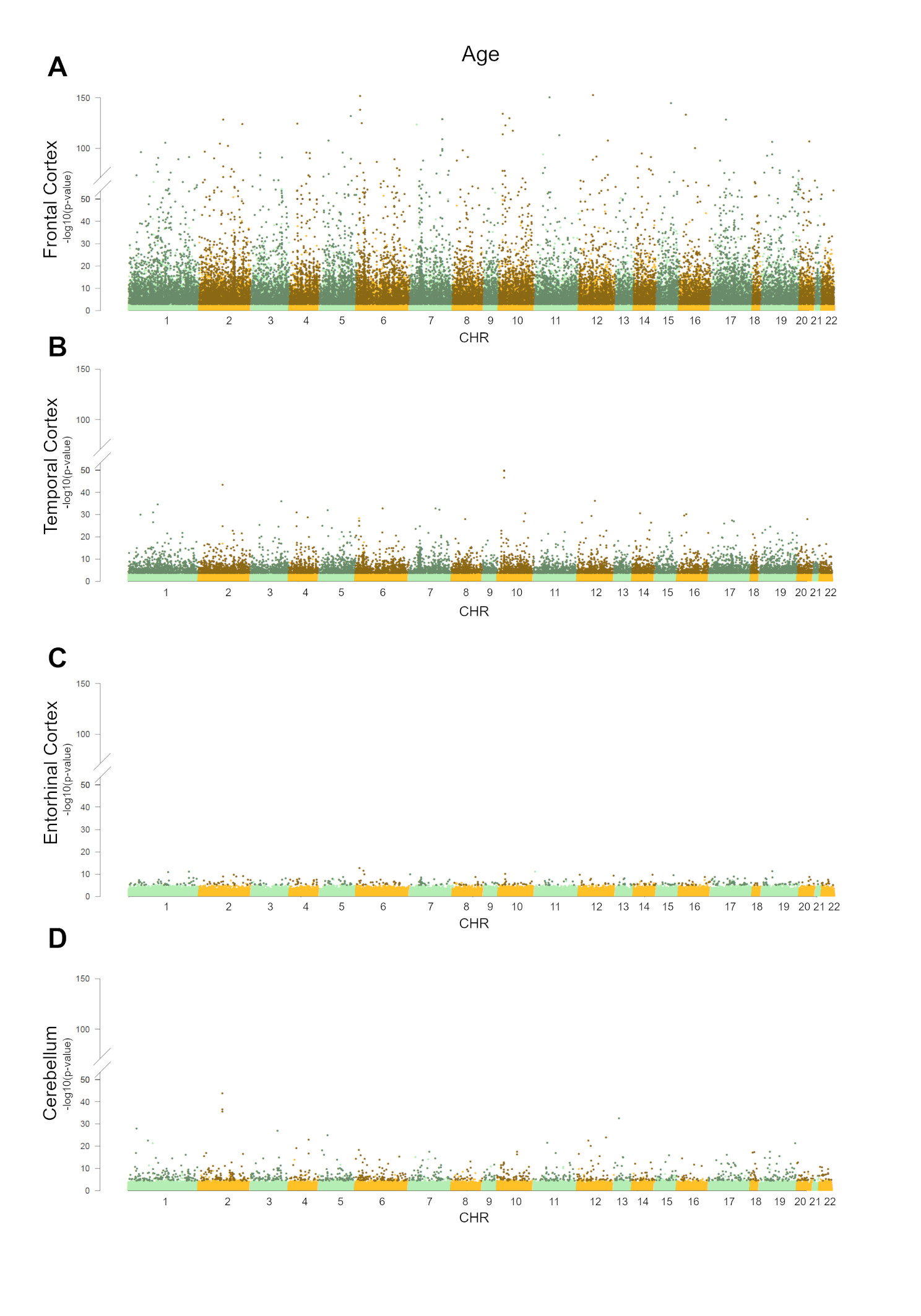

Supplement: Supplementary Figure 3 — Manhattan plots of aDMPs in the four brain regions. The figure displays the Manhattan plots resulting from the meta-analysis of age-associated probes in FC (A), TC (B), ERC (C), and CRB (D). Significant aDMPs are marked with dark color. Scale change across 50 is indicated by an axis break. [file Image_3.jpg]

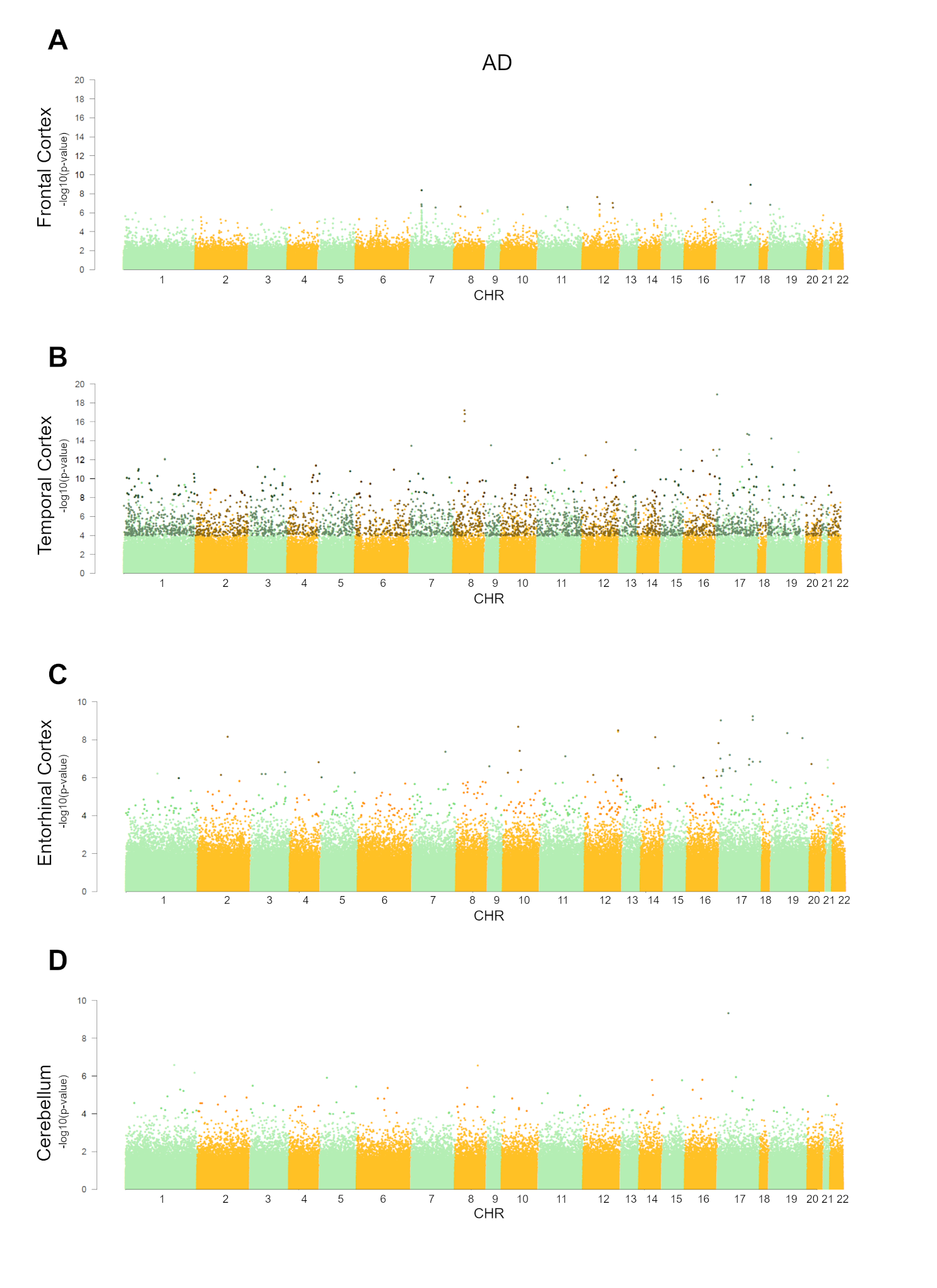

Supplement: Supplementary Figure 4 — Manhattan plots of AD-DMPs in the four brain regions. The figure displays the Manhattan plots resulting from the meta-analysis of AD-associated probes in FC (A), TC (B), ERC (C), and CRB (D). Significant AD-DMPs are marked with dark color. [file Image_4.TIF]

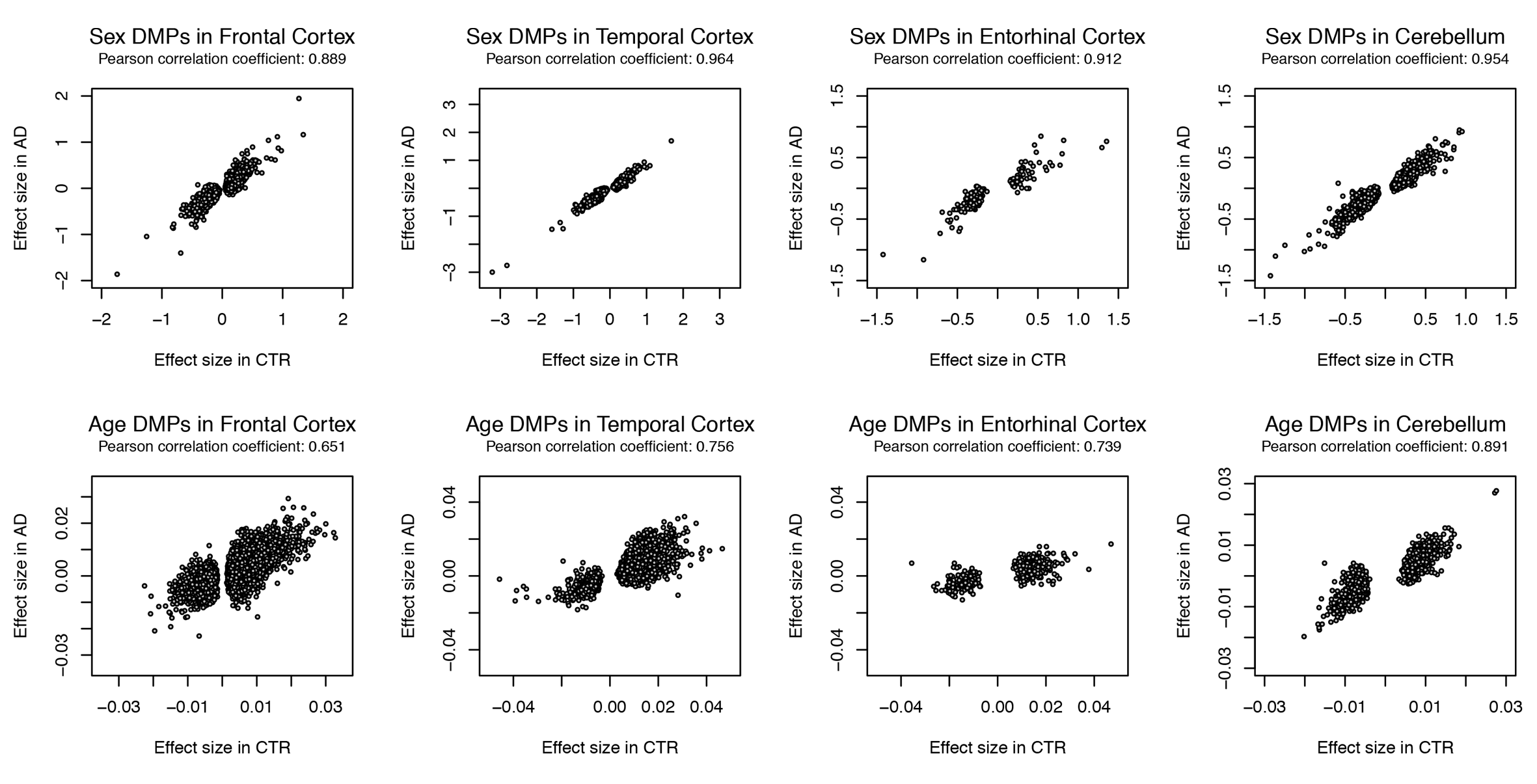

Supplement: Supplementary Figure 5 — Confirmation of sDMPs and aDMPs in AD patients. The scatter plots report, for the sDMPs and aDMPs identified in each tissue in healthy subjects, the effect sizes obtained in healthy subjects against the effect sizes resulting from the meta-analysis in AD patients. Pearson Correlation coefficient is reported in each plot. [file Image_5.TIF]

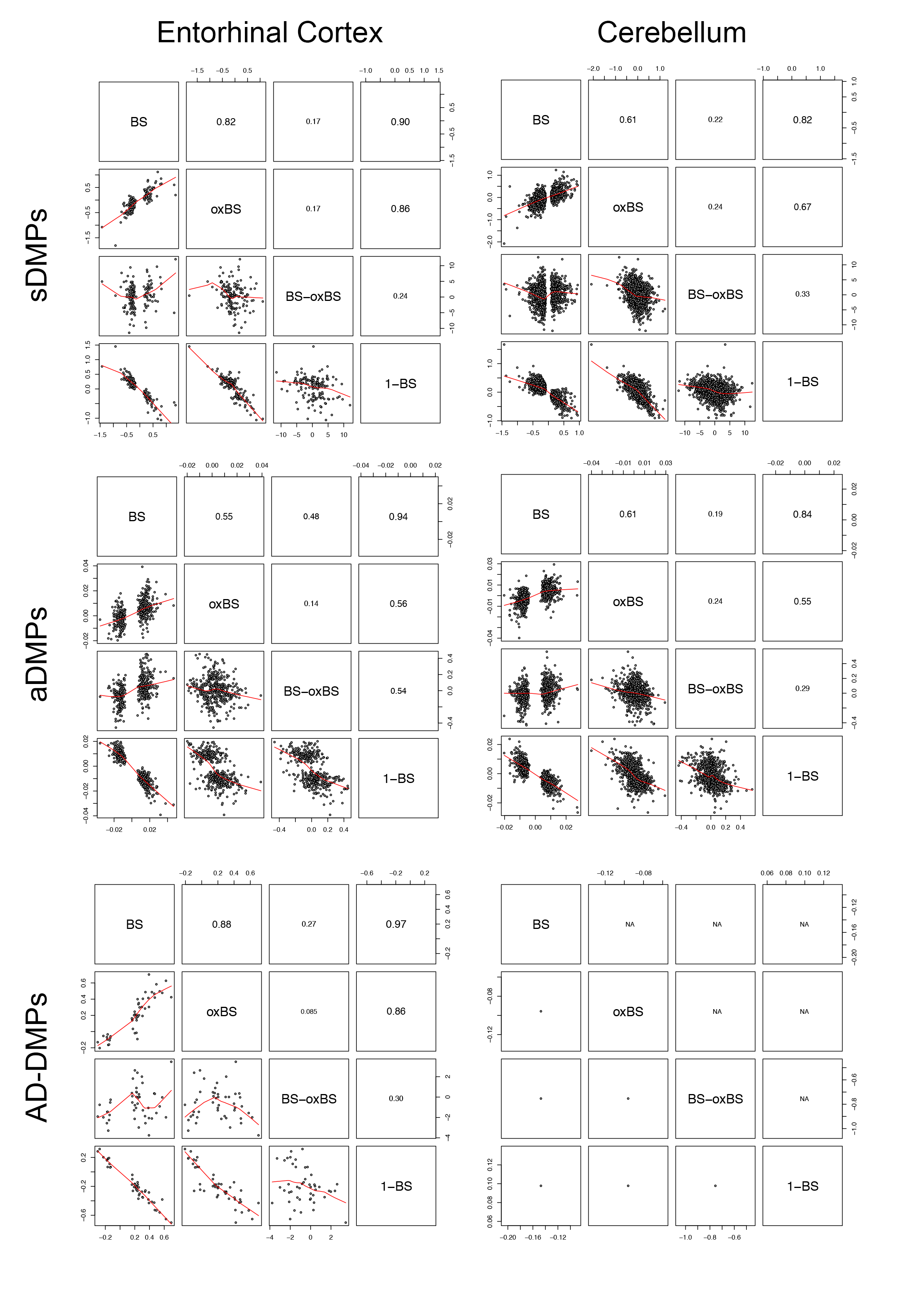

Supplement: Supplementary Figure 6 — Contribution of 5hmC to the epigenetic changes across sex, age, and AD. Correlation plots of the effect sizes of sDMPs, aDMPs, and AD-DMPs identified in ERC and CRB, calculated using BS values (5mC+5hmC), oxBS values (5mC), BS-oxBS values (5hmC), and 1-BS values (5uC). Absolute correlation values are reported. [file Image_6.TIF]
